# Mineralogical and Microstructural Characterization of Historical Masonry Materials from the East Gate of the Shouxian County City Wall

**DOI:** 10.3390/ma19204339

**Published:** 2026-10-15

**Authors:** Mingyi Zhao, Huiyu Li, Jingwei Liang, Qingnian Deng, Jinjing Guo, Muyao He, Liyan Tao, Yile Chen, Liang Zheng

**Affiliations:** 1 School of Digital Art and Design, Qingdao City University, 79 Tiejishan Road, Chengyang District, Qingdao 266000, China; mingyi.zhao@qdc.edu.cn (M.Z.); huiyu.li@qdc.edu.cn (H.L.); jinjing.guo@qdc.edu.cn (J.G.); 2 Faculty of Humanities and Arts, Macau University of Science and Technology, Avenida Wai Long N°S 100–460, Taipa, Macau 999078, China; 2250000402@student.must.edu.mo (J.L.); 2250000398@student.must.edu.mo (Q.D.); muyao.he@network.rca.ac.uk (M.H.); 3 School of Design, Royal College of Art, Kensington Gore, South Kensington, London SW7 2EU, UK; 4 Department of Chemical Engineering, Imperial College London, Exhibition Road, London SW7 2AZ, UK; liyan.tao22@imperial.ac.uk

**Keywords:** ancient city wall, historical masonry materials, polarizing microscope, X-ray diffraction, SEM-EDS, architectural heritage restoration

## Abstract

Characterizing material variation is relevant to the conservation of historic city walls. This study examined five masonry fragments from the East Gate of the Shouxian County City Wall using petrographic microscopy, X-ray diffraction (XRD), and scanning electron microscopy–energy-dispersive spectroscopy (SEM–EDS). Six thin sections showed fine-grained continuous matrices in Samples A and C, abundant coarse grains in B and E, and contrasting optical characteristics between the two sections of D. Calcite and quartz were identified in all five XRD patterns. Local EDS analyses recorded mixed elemental distributions and regions with prominent Fe, Ca, or C and O signals. SEM revealed blocky, platy, and granular particles, together with filamentous, tubular, and folded film-like features. Material heterogeneity here refers to contrasting matrix–grain textures and local elemental distributions within a sample set sharing calcite and quartz. These differences may reflect variation in materials used during construction or repair, without identifying specific historical phases. They support recording individual masonry units and their interfaces before conservation interventions.

## 1. Introduction

### 1.1. Research Background

Architectural heritage encompasses multiple values, including irreplaceable history, regional culture, and construction techniques [1]. Building materials serve as the key physical carriers for preserving this information. The functionality and durability of heritage buildings largely depend on the inherent characteristics of the building materials used and the techniques employed during the construction process [2]. In ancient China, as well as globally, city walls functioned as the primary defensive structures and held significant military and social value throughout a long historical period [3]. Research on defensive architectural heritage worldwide has identified mud/clay, gypsum, and lime mortars as the three most commonly used binding materials prior to the invention of Portland cement [4]. Chinese traditional concrete, known as “San-he-tu,” first appeared in the Song Dynasty text *Ying Zao Fa Shi*. This type of lime mortar is created by mixing lime, sand, and soil in specific ratios through traditional Chinese methods [5]. As a composite building material, it exhibits strong adhesion and good durability, making it widely used in constructing ancient city walls.

Historical Chinese city walls contain masonry materials with different aggregates and binding components. Studies of the Xindeng city wall describe lime mortar with sand and locally incorporated brick or clay fragments [6]. Research on the Hezhou and Kaifeng city walls also reports on the use of sticky-rice-containing mortars [7,8]. These examples highlight the need to examine mineral constituents, grain characteristics, and matrix textures together when characterizing historical city-wall materials.

### 1.2. Literature Review

Historic masonry can be affected by climatic exposure, moisture transport, and freeze–thaw processes [9,10,11]. Wall surfaces also provide habitats for urban vegetation [12], while surrounding planting can modify the microclimate experienced by historic masonry [13]. Conservation interventions require case-specific decisions [14], and inappropriate repair materials can contribute to deterioration [15].

The study of historical brick masonry materials usually requires a combination of techniques such as polarized light microscopy, XRD, SEM-EDS, thermal analysis, and porosity measurements. For example, thin section observation can describe aggregate particle size, sorting, matrix continuity, and reaction boundaries; XRD confirms bulk crystalline minerals; SEM reveals particle surface and local morphology; and EDS provides the elemental composition of a single field of view [16,17]. López-Arce et al. distinguished the raw material and manufacturing differences in Toledo historical bricks through multi-technical combined analysis [16]. The experimental firing studies of Cultrone et al. showed that the carbonate–silicate reactions and the porosity evolution are controlled by the raw material minerals, the firing temperature, and the firing atmosphere [18,19].

A comparative framework at different scales has been developed to investigate the material history of Chinese historic bricks. Shu et al. compared the minerals, petrography, physical properties, and maximum firing temperature of Shanghai bricks in the 19th and 20th centuries and revealed the changes in raw materials and microstructure during the transition from traditional bricks to modern processes [20]. Li et al. undertook XRF, XRD, and microscopic investigations of Ming and Qing dynasty bricks of the Xi’an city wall, associating quartz, feldspar, and a minor amount of calcite with refining and firing of the raw material [21]. Shi et al. compared the pore structures of ancient city-wall bricks using multiple analytical methods, showing that the measured pore characteristics depend on the method employed [22].

There are scale differences between elemental composition and mineral phase. Mishra et al. compared raw materials and production technologies using the geochemical characteristics of historical bricks, emphasizing that major and trace elements need to be interpreted in conjunction with mineral phase and origin background [23]. For powder samples, a single EDS field of view may fall on iron-rich particles, carbonate aggregates, or surface deposits and cannot be equivalent to the chemical composition of the whole sample. Local EDS observations complement the bulk crystalline-phase information from XRD and the textures observed in thin sections.

Historical brick masonry systems also include mortar, grouting, and finishing materials. Elsen pointed out that optical microscopy is the basic method for distinguishing ancient mortar binders, aggregates, and reaction boundaries [17]. Calcium carbonate-starch composites have been used in some ancient Chinese brick mortars, as confirmed by Yang et al. and Xiao et al. [24,25]. Studies of glutinous rice mortar combine mineralogical characterization with tests for starch-containing organic components [24,25]. Wei et al. identified aerial lime mortars in the sampled city-wall joints in Anhui, whereas a separate Song-dynasty tomb sample contained a pozzolanic mortar with lime, clay, sand, and cocciopesto-like material [26], indicating that the historical masonry materials in Anhui themselves had significant type differences.

Research on environmental weathering has shown that soluble salts, wet-dry cycles, acid rain, and microbial activity can induce mineral transformation, particle loosening, and secondary coatings on the surface of bricks [27,28]. Zhao et al. conducted a metagenomic study on the biofilm on the surface of the Shouxian County city wall and identified the main groups, including *Proteobacteria*, *Actinobacteria*, *Cyanobacteria*, *Bacteroidetes*, and *Firmicutes*, and a small number of *Ascomycota* fungi [29]. The study also pointed out that actinomycetes can form hyphal-like coverings, and microbial extracellular polymers can adhere to airborne particles and pollutants; the community differences in different wall sections are related to the surrounding human, animal, and plant activities and geochemical environment [29]. These results indicate that the surface of Shouxian County bricks has conditions for biofilm formation and also suggest that filamentous or film-like SEM objects need to be interpreted separately from inorganic minerals.

### 1.3. Problem Statement and Objectives

**Figure 1:**
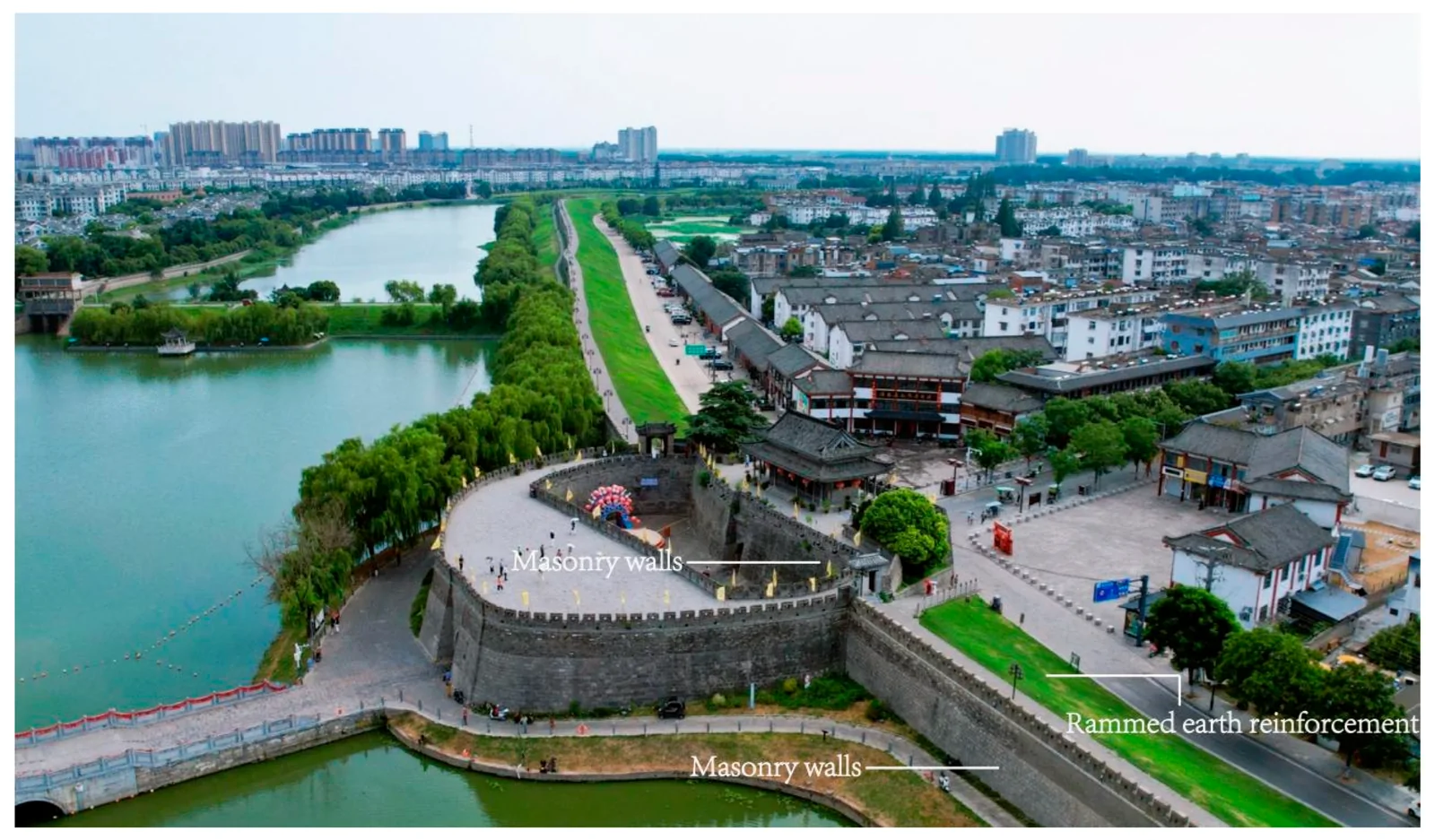
The structure of the “Sloped-Ramp Fortified City”. (Image source: Photo by the author).

Before the Ming Dynasty, the construction of city walls in the north and south of China differed. The north mostly used traditional rammed earth city walls, while the south favored brick and stone city walls [30]. However, the Shouxian County city wall, located at the junction of northern and southern China, consists of both a rammed earth structure and a brick and stone structure. The outer side of the city wall is brick and stone, while the inner side is reinforced with rammed earth, forming a unique “Sloped-Ramp Fortified City” appearance (Figure 1). The existing Shouxian County city wall dates from the earliest Song Dynasty and has undergone multiple repairs from the Ming and Qing Dynasties to the present day. It is preserved to this day with diverse building materials and complex environmental impacts.

The relationship between mineral assemblages and petrographic textures remains insufficiently characterized by the materials at the East Gate. This study addresses three questions: which crystalline phases occur in the selected fragments, how their matrix–grain textures differ, and how local elemental heterogeneity relates to these material differences. Petrographic microscopy, XRD, and SEM–EDS are combined to examine these questions across the five sampled fragments.

## 2. Materials and Methods

### 2.1. Study Area: The East Gate and Its Barbican of Shouxian City Wall

**Figure 2:**
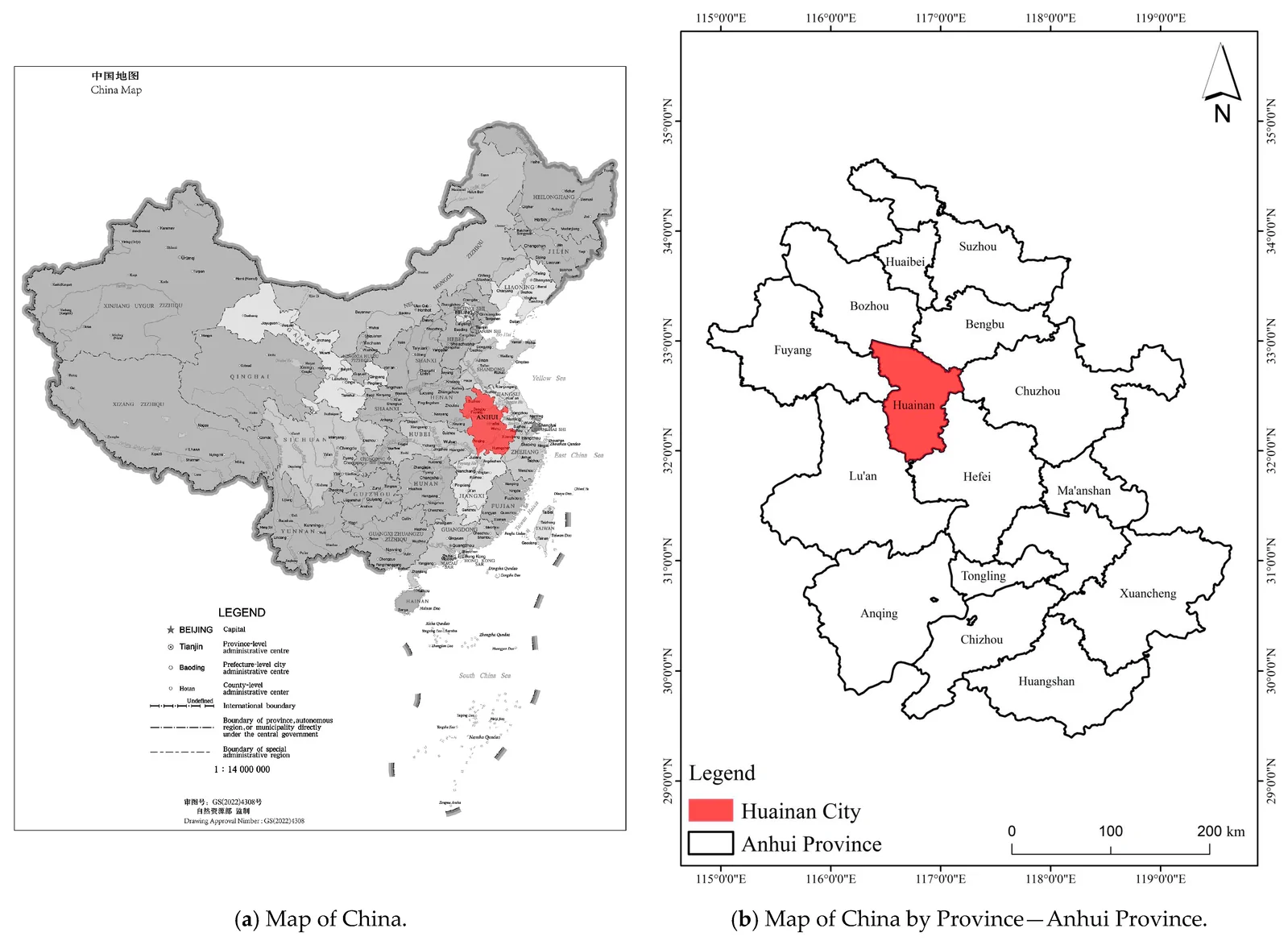

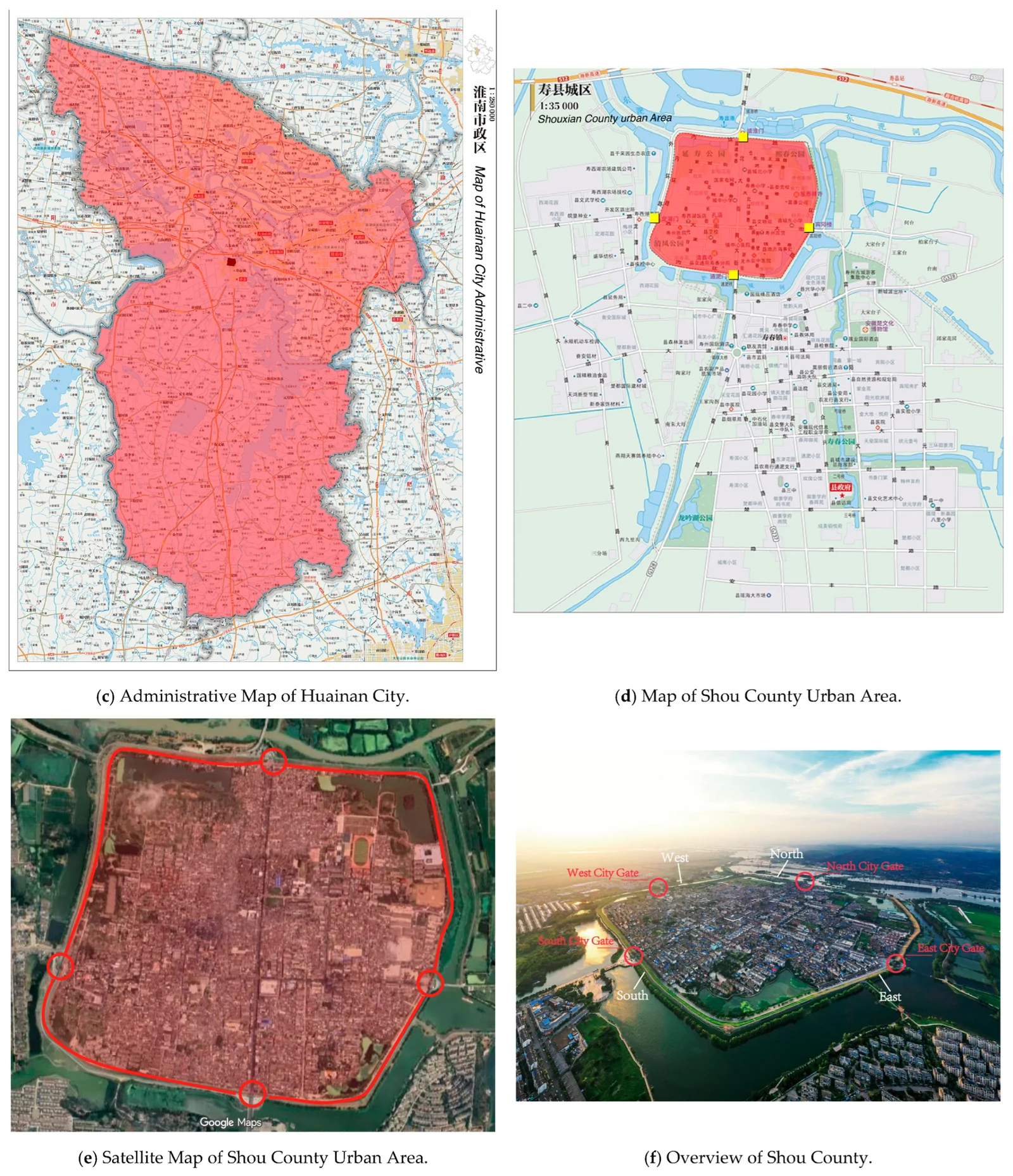
Location of the Shou County City Wall. (**a**) Map of China; (**b**) Map of China by Province—Anhui Province; (**c**) Administrative Map of Huainan City; (**d**) Map of Shou County Urban Area; (**e**) Satellite Map of Shou County Urban Area; (**f**) Overview of Shou County. (Image source: by the author).

Shouxian County is in the suburbs of Huainan City, Anhui Province, in East China (31°54′ N, 116°40′ E) (Figure 2). It belongs to the northern subtropical humid monsoon climate. The average temperature in January is 0.7 °C, the average temperature in July is 27.9 °C, and the average annual precipitation is 906 mm (Appendix D). Because it is adjacent to the Huai River, it has been flooded many times in history. According to the Shouxian County Annals, Shouxian County experienced flooding approximately every five years between 1671 and 1949. The city—referring to the entire city of Shouxian, including its four walls, rather than just the East Gate and its barbican where the sampling took place—underwent repairs nearly thirty times during the Ming and Qing dynasties alone. After the 45th year of Jiajing in the Ming Dynasty (1566 AD), the floods basically only surrounded the outer city wall, and there was almost no flooding inside the city [31].

**Figure 3:**
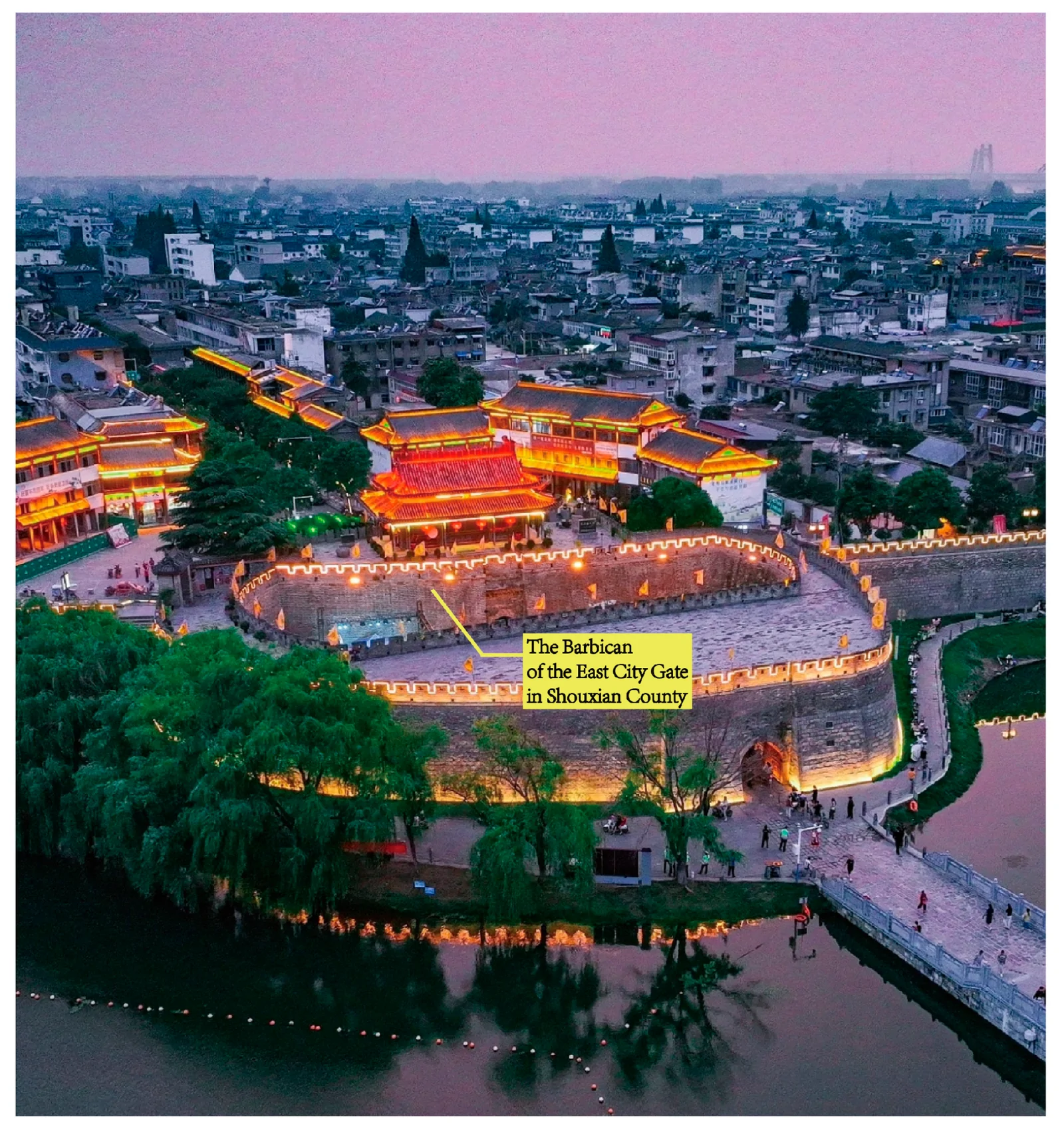
The Barbican of the East City Gate in Shouxian County. (Image source: by the author).

**Figure 4:**
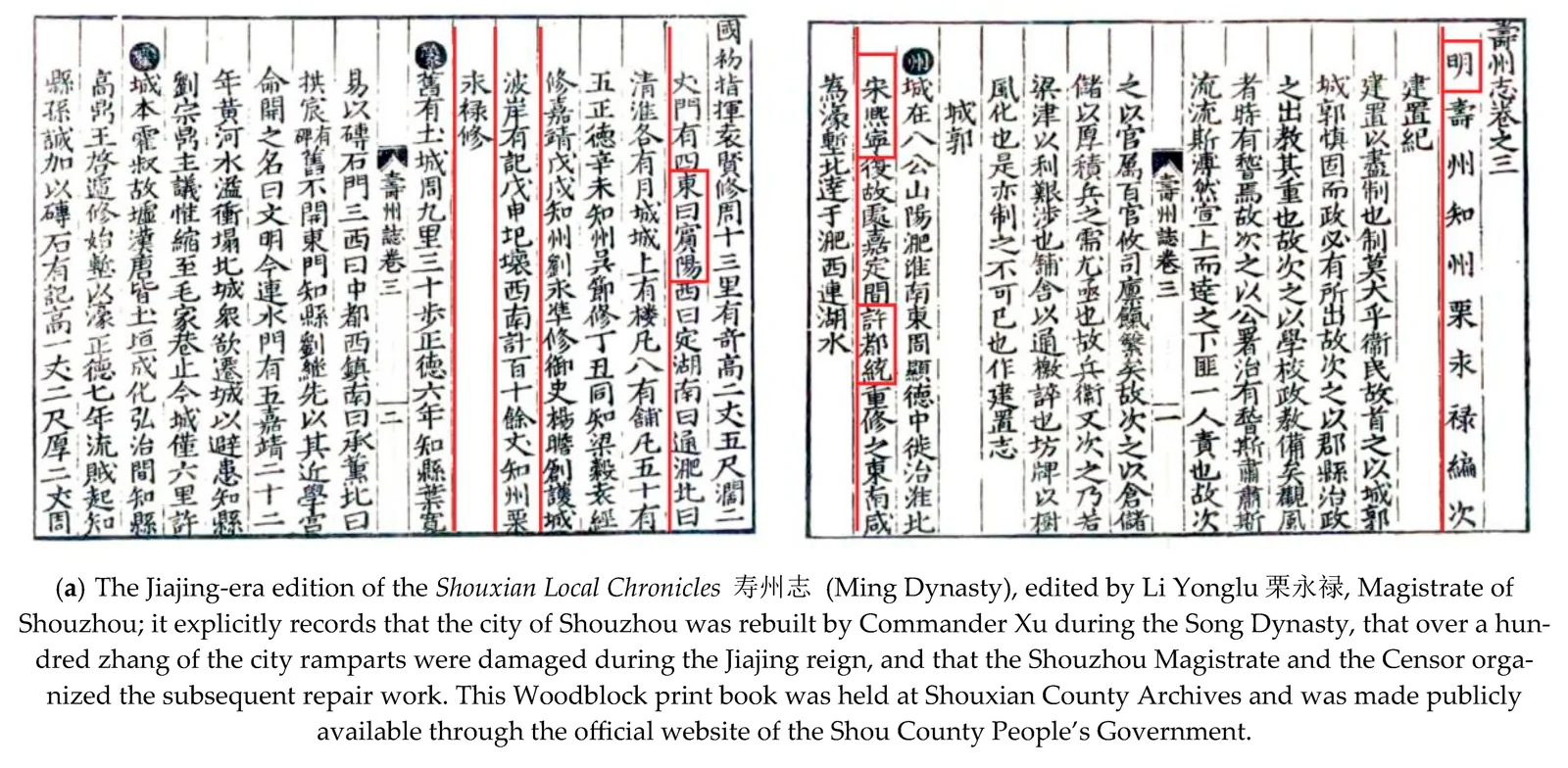

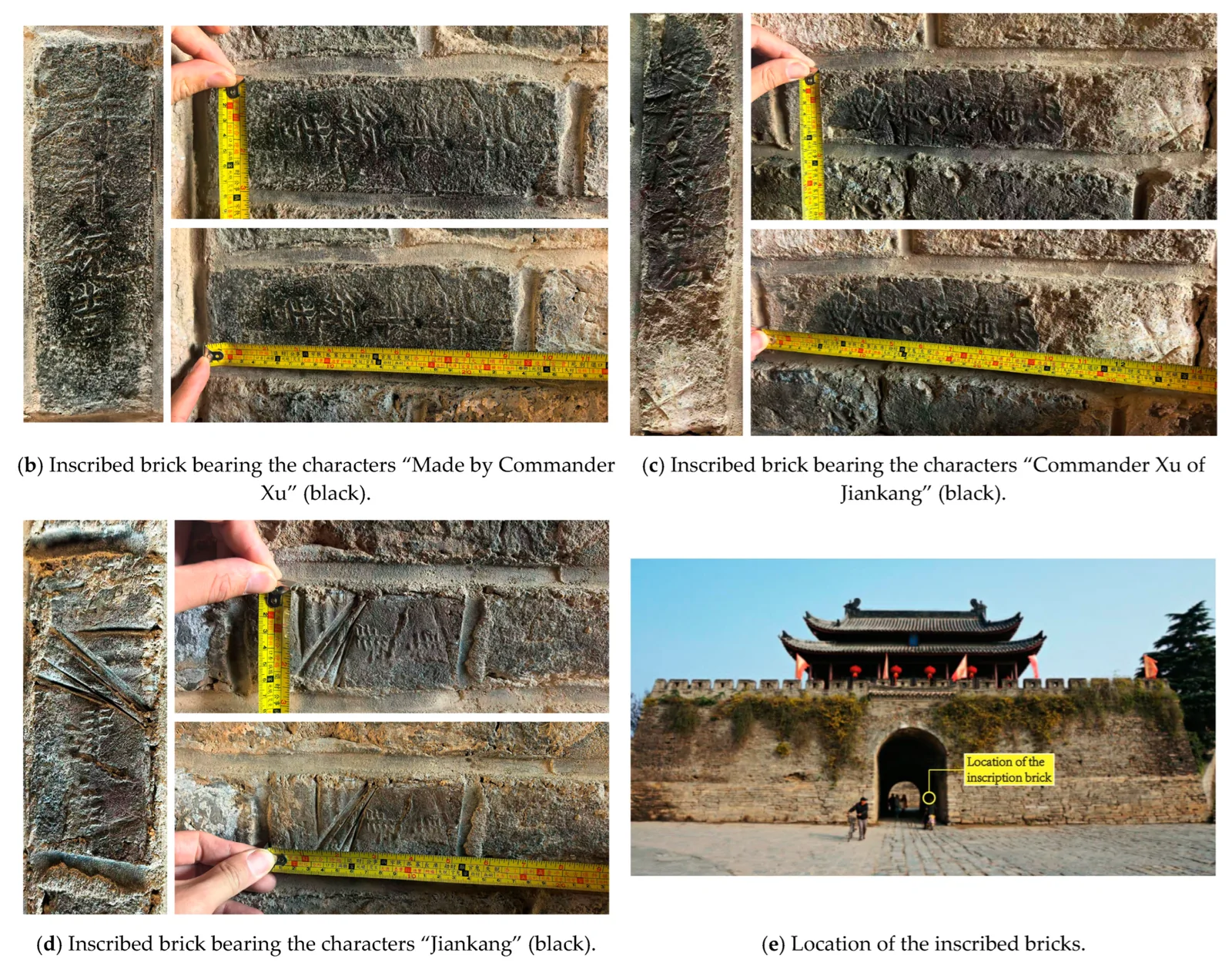
The Barbican of the East City Gate in Shouxian County. (**a**) The Jiajing-era edition of the *Shouxian Local Chronicles* (Ming Dynasty), compiled by Li Yonglu, Magistrate of Shouzhou [31]; it explicitly records that the city of Shouzhou was rebuilt by Commander Xu during the Song Dynasty, that over a hundred zhang of the city ramparts were damaged during the Jiajing reign, and that the Shouzhou Magistrate and the Censor organized the subsequent repair work. (**b**) Inscribed brick bearing the characters “Made by Commander Xu” (black). (**c**) Inscribed brick bearing the characters “Commander Xu of Jiankang” (black). (**d**) Inscribed brick bearing the characters “Jiankang” (black). (**e**) Location of the inscribed bricks. (Image source: by the author).

The ancient city wall of Shouxian still retains the basic appearance of being enclosed on all four sides. It has four city gates in the east, south, west, and north, and each is equipped with a barbican (Figure 3). The oldest existing part of the Shouxian city wall is the city wall of the east gate and its barbican (the sampling point of this study). It was built in the twelfth year of Emperor Ningzong of the Southern Song Dynasty (1206 AD) and was constructed under the supervision of Xu Jun, the then governor of Jiankang. It was extensively repaired during the Ming and Qing Dynasties, and some protective restoration was carried out in modern times [32] (Figure 4). The city wall has a circumference of 7141 m and a height of 8.3 m.

### 2.2. Sampling and Sample Number

**Figure 5:**
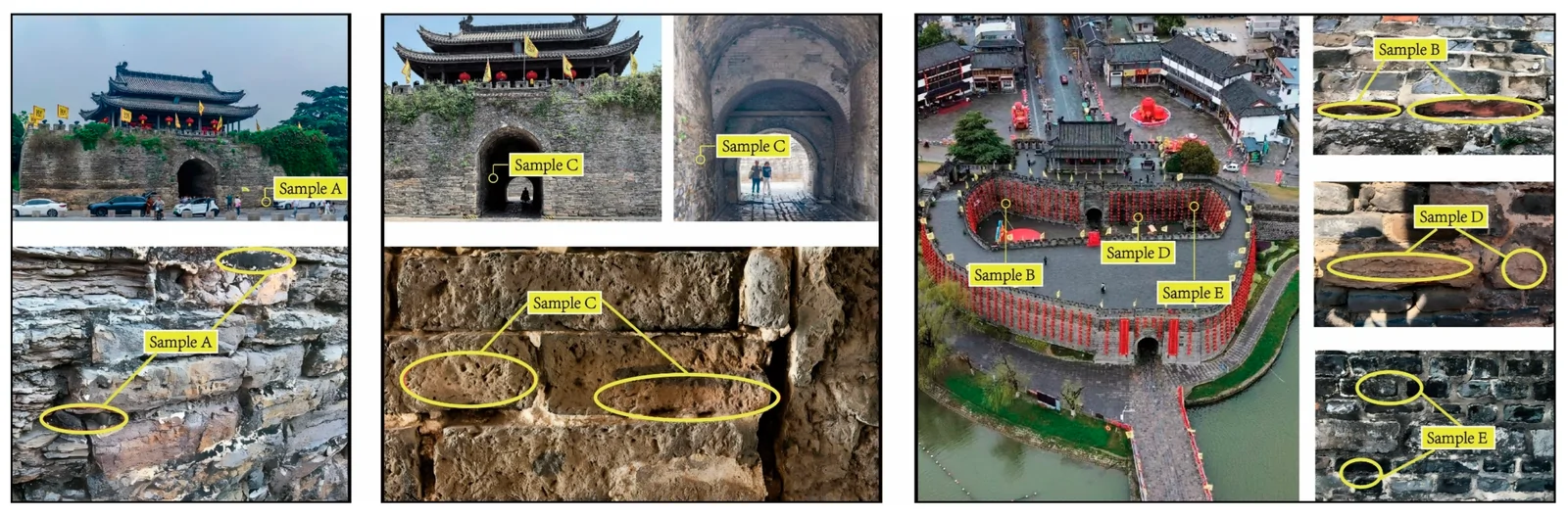
Sampling locations and the appearance of the city wall. (Image source: Photo by the author).

**Figure 6:**
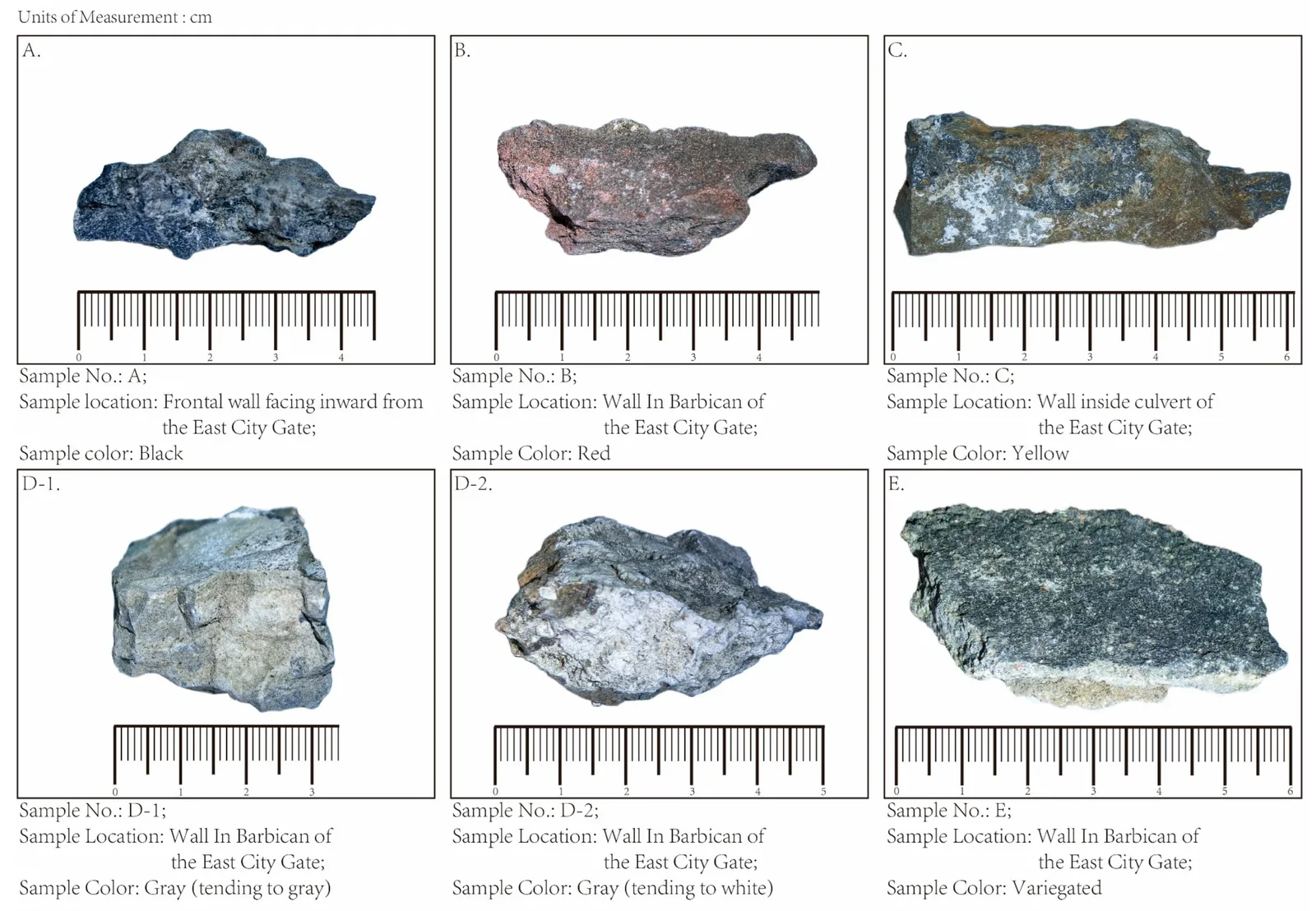
Appearance and dimensions of the sample. (Image source: by the author).

Five masonry fragments (A–E) were collected from the East Gate and its barbican to compare locations with contrasting surface colors and masonry appearances (Figure 5 and Figure 6). Sample A, a black masonry fragment, was collected near ground level from the city-facing side of the East Gate adjacent to the residential area. Sample B, a red fragment, was collected from the inner wall on the right side of the East Gate barbican. Sample C, a yellow fragment, was collected from the inner wall of the East Gate culvert. Sample D, a gray fragment with whitish areas, and Sample E, a multicolored fragment, were collected from the inner wall on the left side of the barbican. Sampling was restricted to loosing near-surface material and involved no deep excavation (Figure 5). The location of A is adjacent to a residential area, and C is situated within the shaded gate culvert.

XRD and SEM–EDS were performed on Samples A–E. Two thin sections, D-1 and D-2, were prepared from the grayish and whitish parts of Sample D, respectively. Together with one thin section from each of A, B, C, and E, this yielded six thin sections. Table 1 summarizes the sampling locations and surface appearances.

### 2.3. Experimental Procedure and Parameters

Six thin sections approximately 30 μm thick were examined at room temperature using an Olympus GX71 microscope (Olympus, Tokyo, Japan) in transmitted-light mode under plane-polarized light (PPL) and cross-polarized light (XPL). The objectives were 4×, 10×, 25×, 50×, and 100×, with archived images covering scales of 500, 200, 100, and 50 μm. The 222 archived images were examined in PPL/XPL pairs to describe matrix continuity, grain size and sorting, optical characteristics, and visible fractures. Dark regions in unstained thin sections may simultaneously contain pores, opaque phases, or a dark matrix; therefore, porosity was not directly calculated.

XRD was performed using a Rigaku SmartLab SE diffractometer (Rigaku Corporation, Akishima, Japan). Powder samples were subjected to Cu Kα radiation at a wavelength of 1.54060 Å, a scanning range of 10–80° (2θ), and a scanning speed of 2°/min. This paper reviews the original diffraction data, peak tables in the test report, and matching diagrams of the supporting materials, using reference peaks for calcite (PDF 00-005-0586), quartz (PDF 00-046-1045), dolomite (PDF 00-036-0426), and albite (PDF 00-009-0466). XRD was used for qualitative phase identification. Approximate d-spacings were calculated from the reported 2θ positions using Bragg’s law and a wavelength of 1.54060 Å.

Particle morphology was examined using a Sigma 360 field-emission scanning electron microscope (ZEISS, Oberkochen, Germany). Powder specimens for SEM–EDS were prepared by grinding portions of the masonry fragments remaining after thin-section preparation. Powder was directly fixed to conductive adhesive, sputtered with gold, and observed in secondary electron mode. The accelerating voltage for morphology images was 3.0 kV, and the archiving magnifications were 50×, 100×, 200×, 500×, 1000×, 2000×, 5000×, and 10,000×, with working distances of approximately 5.5–8.9 mm. Each sample was archived with 14 SEM images: one field of view each at 50× and 100×, and two fields of view each at 200–10,000×, for a total of 70 images.

EDS elemental mapping was performed at 15.0 kV and 5000×, with a working distance of 6.7–7.9 mm. Table 2 summarizes the parameters, outputs, and usage methods of the three types of experiments.

SEM was used to describe the morphology and aggregation of prepared particles. EDS spectra and maps were evaluated at the scale of the analyzed field, with the reported percentages treated as local semi-quantitative estimates. The recorded carbon signal may include a contribution from the conductive adhesive.

Bulk crystalline phases were identified by XRD, and matrix–grain relationships were described from paired PPL/XPL images. SEM–EDS complemented these observations with particle morphology and local elemental distributions.

The optical dataset comprises 222 images from six thin sections. The SEM dataset comprises 70 images from five powder specimens, with one field per specimen at 50× and 100× and two fields at each magnification from 200× to 10,000×. The reported EDS dataset contains one mapped region per sample, together with its spectrum and numerical output. XRD patterns were examined alongside the corresponding peak tables and phase-matching records.

## 3. Results

### 3.1. Petrographic Analysis

**Figure 7:**
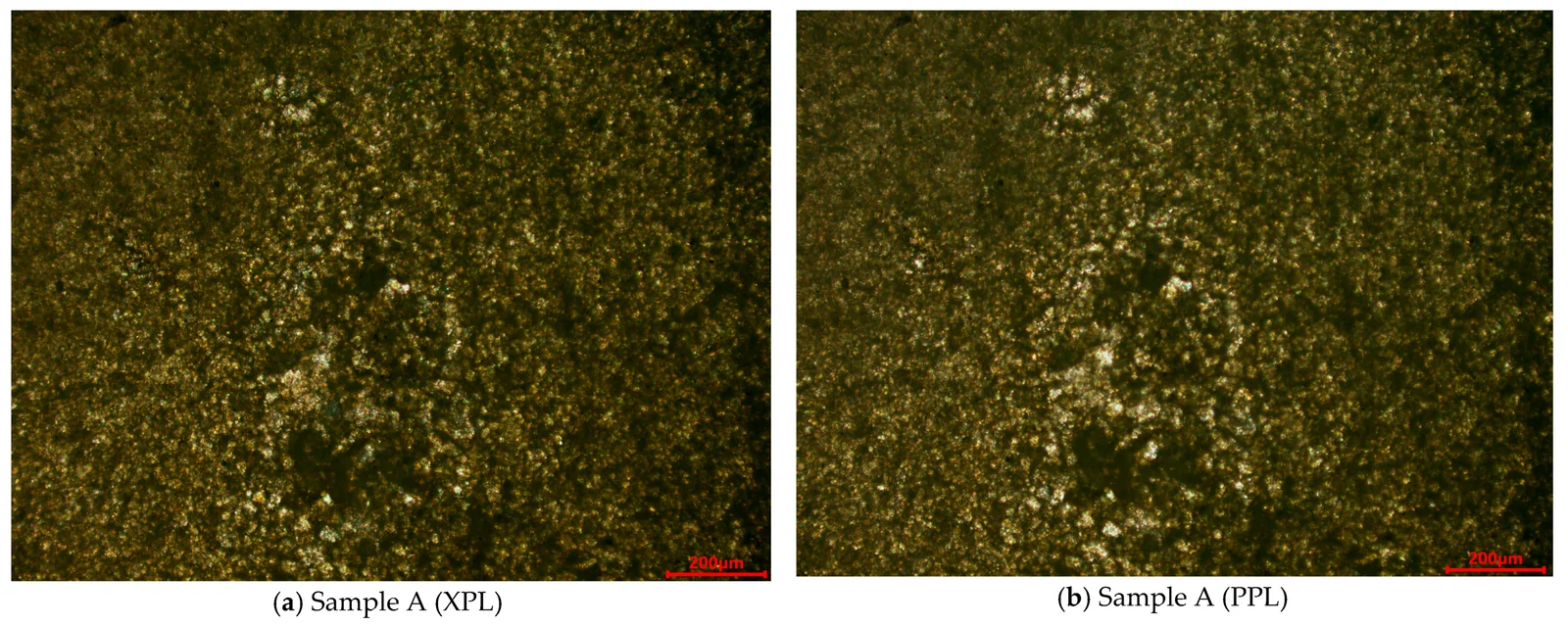

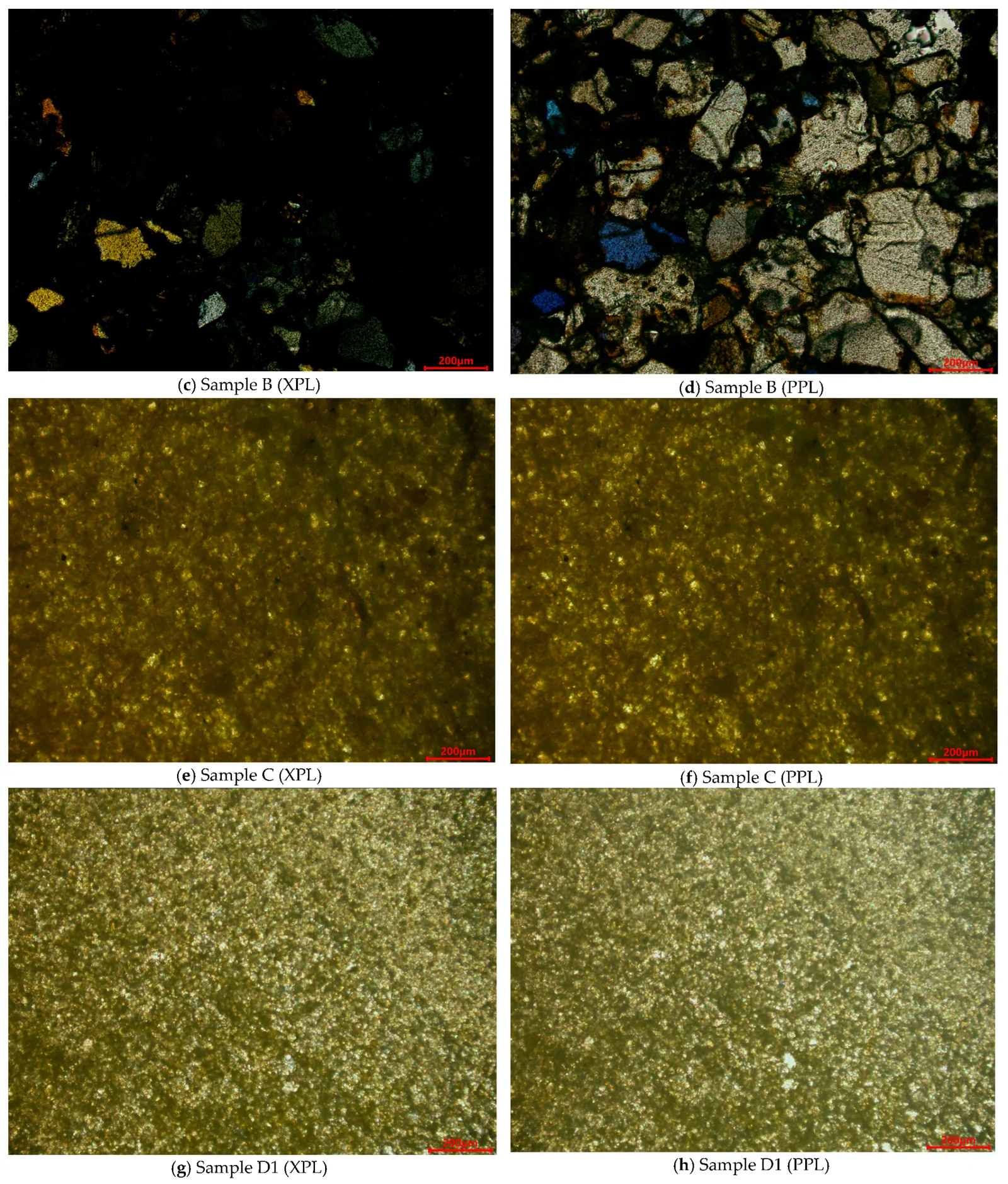

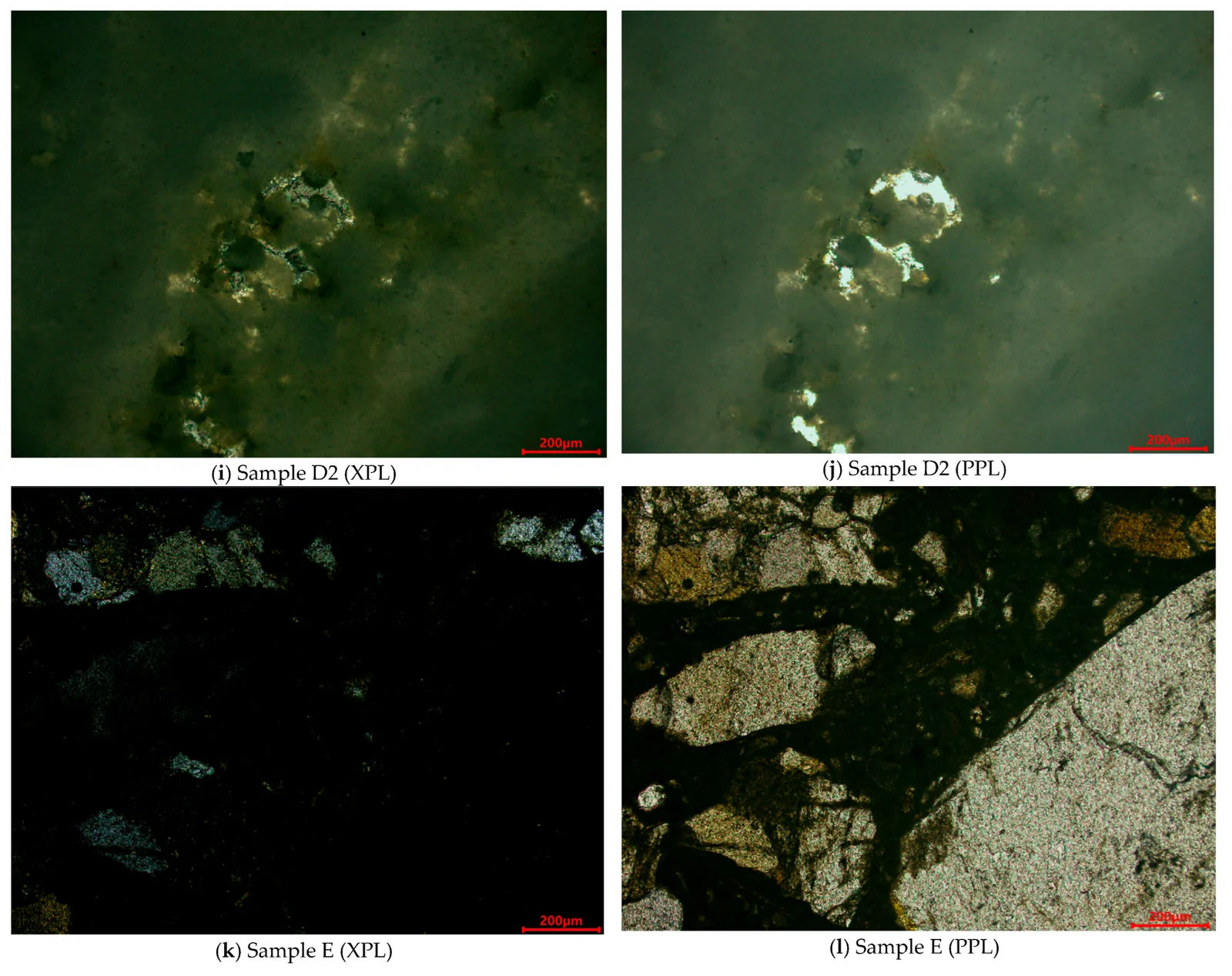
Representative thin-section photomicrographs of Samples A, B, C, D-1, D-2, and E under cross-polarized light (XPL) and plane-polarized light (PPL). Scale bars are shown in each panel.

The six thin sections differ in matrix continuity, coarse-grain abundance, grain shape, and optical characteristics (Figure 7). Samples A and C have fine-grained continuous matrices, B and E contain abundant coarse grains, and D-1 and D-2 have matrix-dominated textures.

Sample A (Figure 7a,b) has a dark brown to olive-colored matrix containing dispersed, sub-angular transparent grains and small grain clusters. Many fine grains display low-order interference colors under XPL. Sample B (Figure 7c,d) contains sub-angular to angular grains within a discontinuous dark matrix. Grain sizes are commonly approximately 100–300 μm and locally exceed 500 μm, indicating poor sorting. Sample C (Figure 7e,f)has a fine-grained continuous matrix with locally visible linear fissures and bright grain clusters. Its fine grains show predominantly low-order interference colors.

Sample D-1 (Figure 7g,h) has a light grayish-yellow matrix with few coarse grains, small dark spots, and narrow fractures. Weak to low-order interference colors occur across much of the section. Sample D-2 (Figure 7i,j) has a light grayish-green translucent matrix with weak birefringence and sparse brighter grain clusters. These optical characteristics distinguish the two sections prepared from Sample D.

Sample E (Figure 7k,l) contains abundant sub-angular to subrounded grains approximately 200–500 μm in size within a dark intergranular matrix. Grains with low-order gray-white and higher interference colors occur together with composite particles. Table 3 summarizes the matrix–grain textures, and the complete image series is presented in Appendix A.

### 3.2. Multi-Scale SEM Particle Morphology

**Figure 8:**
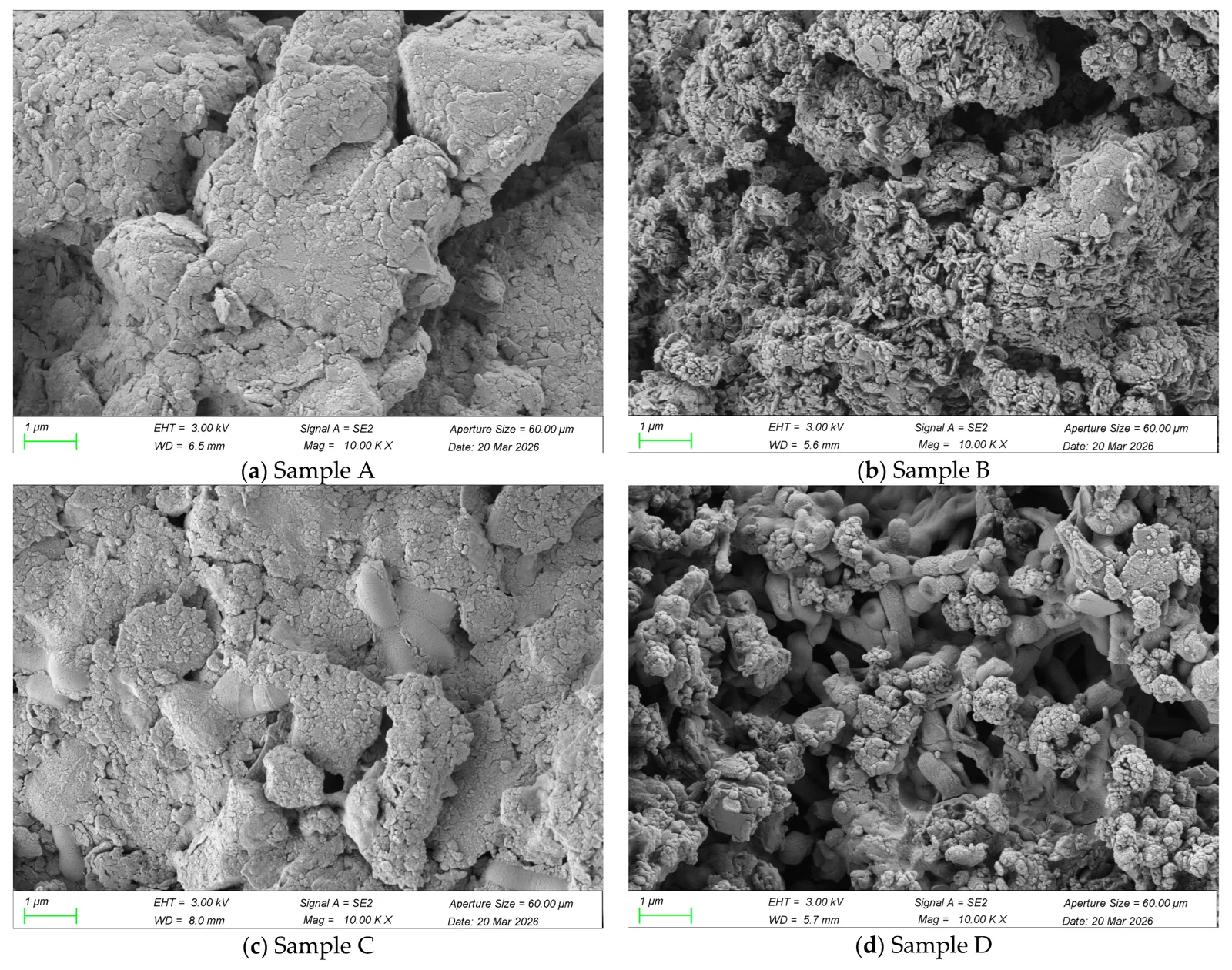

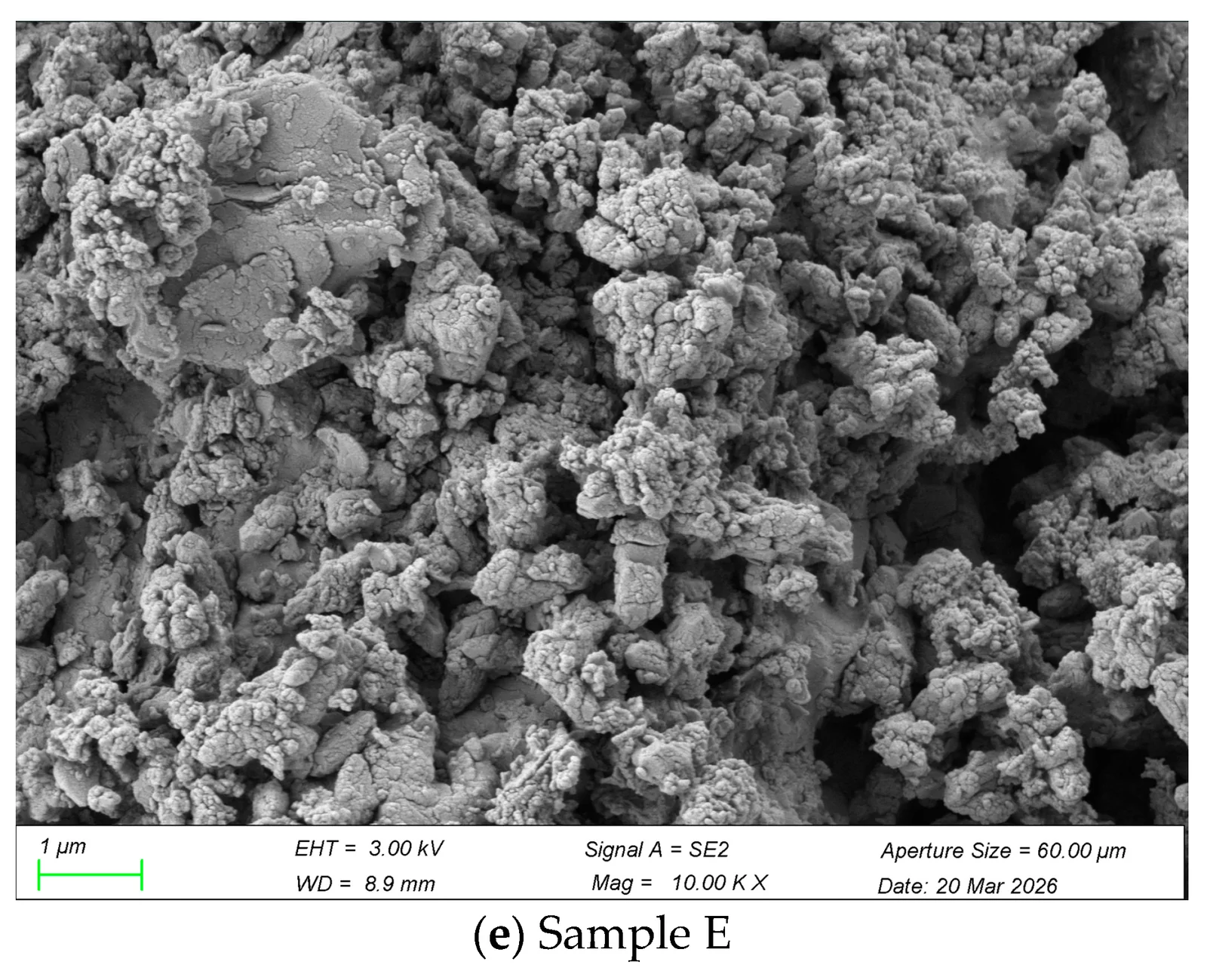
SEM secondary-electron images of Samples A-E at 10,000×. Instrument scale bars and operating conditions are retained in each panel.

SEM images at magnifications from 50× to 10,000× show particle aggregates, individual particles, and local surface features. Figure 8 compares Samples A–E at 10,000×, and images at other magnifications are provided in Appendix B.

At 10,000×, the five powder specimens differ in particle shape, aggregation, and surface morphology (Figure 8). Sample A (Figure 8a) shows stacked blocky fragments with fine particles adhering to their surfaces. A few microcracks are visible within the larger fragments. Sample B (Figure 8b) contains fine, irregular particles with both platy and granular morphologies. Gaps are visible between the particle clusters. Sample C (Figure 8c) shows relatively large blocky aggregates surrounded by finer particles. The boundaries of the larger aggregates remain clearly visible. Sample D (Figure 8d) shows interwoven granular, rod-like, and filamentous features. Fine particles adhere to the larger aggregates. Sample E (Figure 8e) contains aggregates of similarly sized fine granular particles, with gaps between the clusters.

### 3.3. Local Elemental Distributions from EDS Mapping

**Figure 9:**
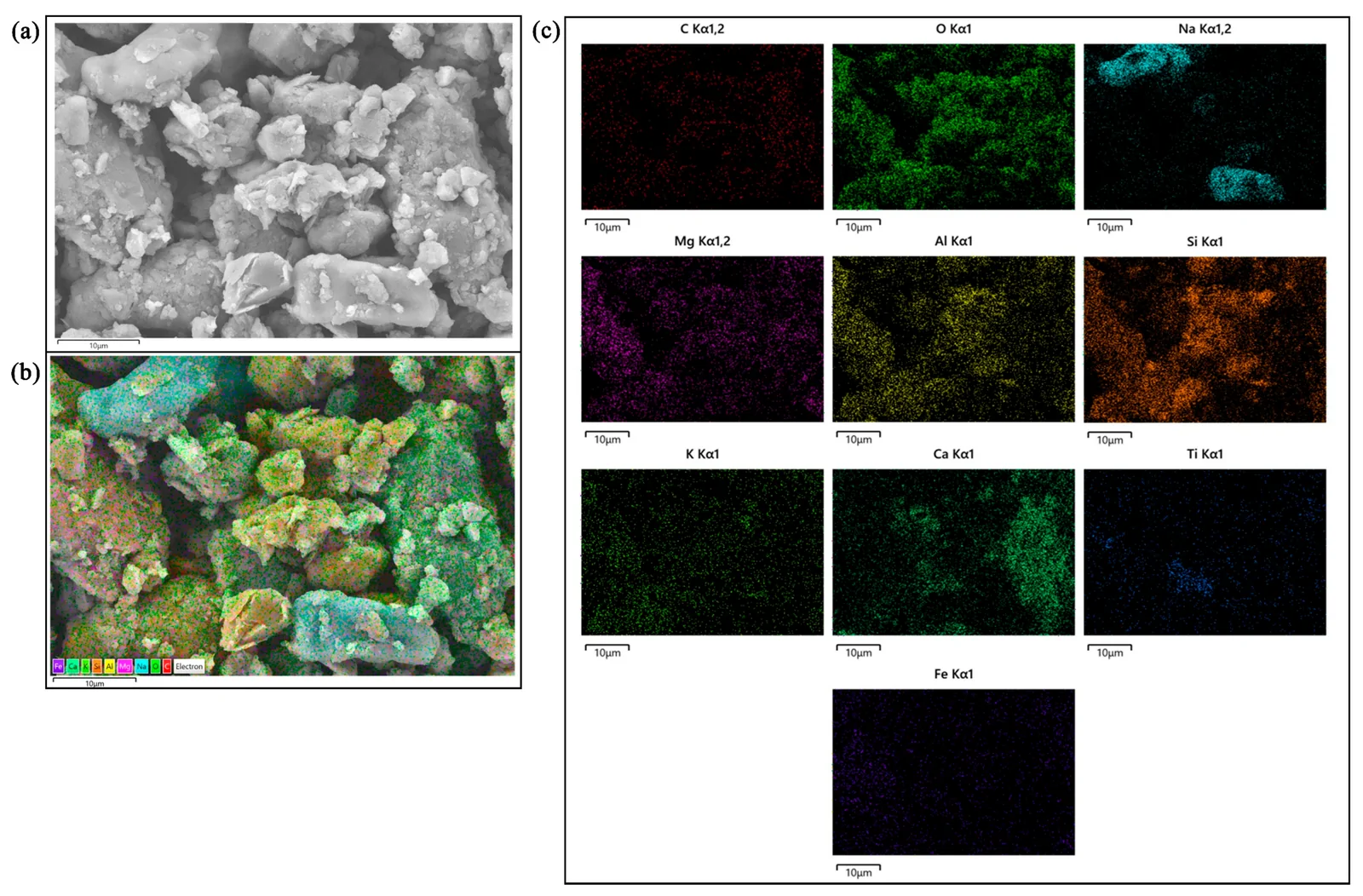
SEM–EDS elemental mapping of Sample A at 5000×. (**a**) Secondary-electron image of the analyzed region. (**b**) Elemental overlay map. (**c**) Individual elemental maps. The corresponding EDS spectrum is provided in Appendix C (Figure A12a).

**Figure 10:**
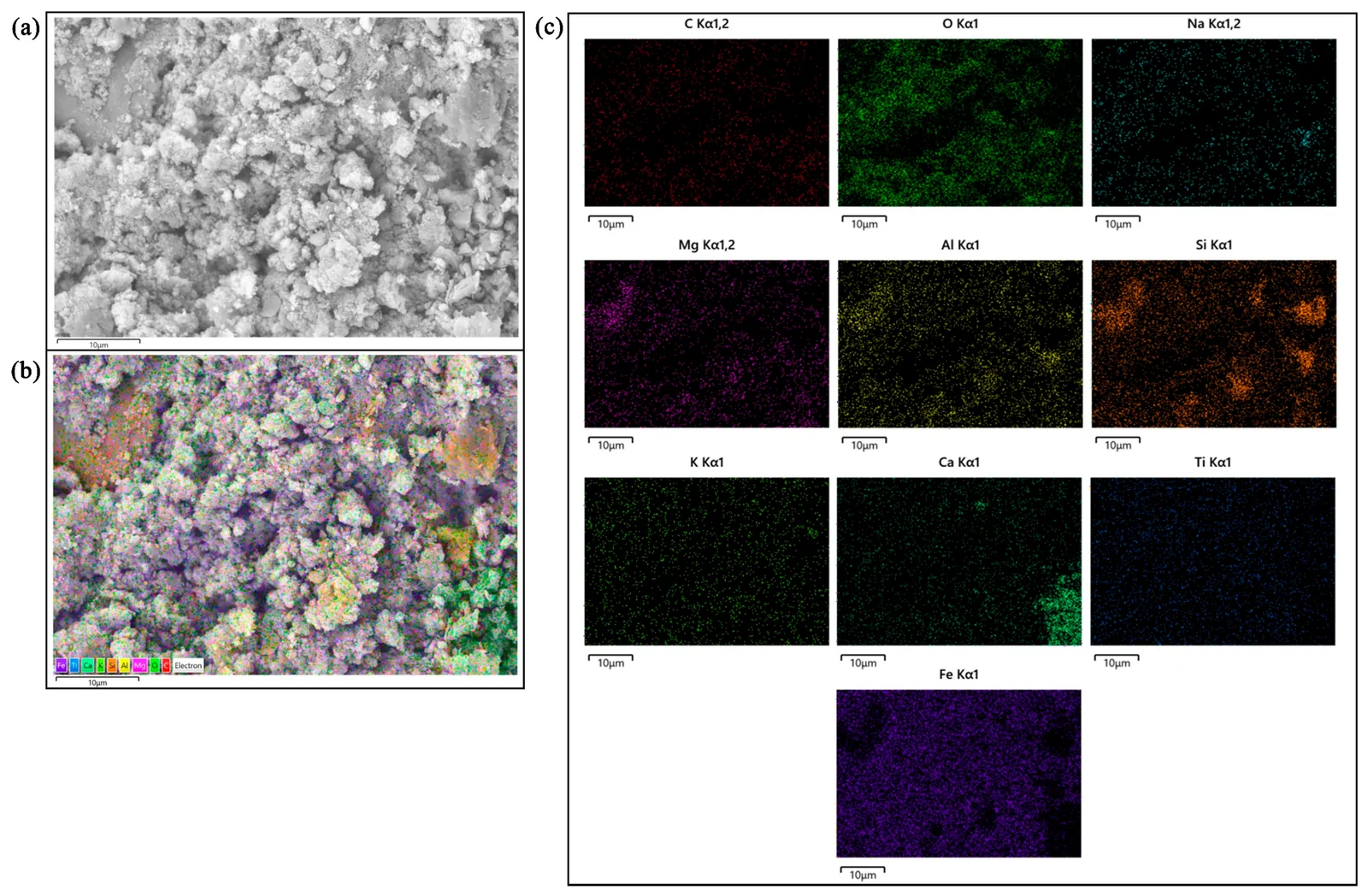
SEM–EDS elemental mapping of Sample B at 5000×. (**a**) Secondary-electron image of the analyzed region. (**b**) Elemental overlay map. (**c**) Individual elemental maps. The corresponding EDS spectrum is provided in Appendix C (Figure A12b).

**Figure 11:**
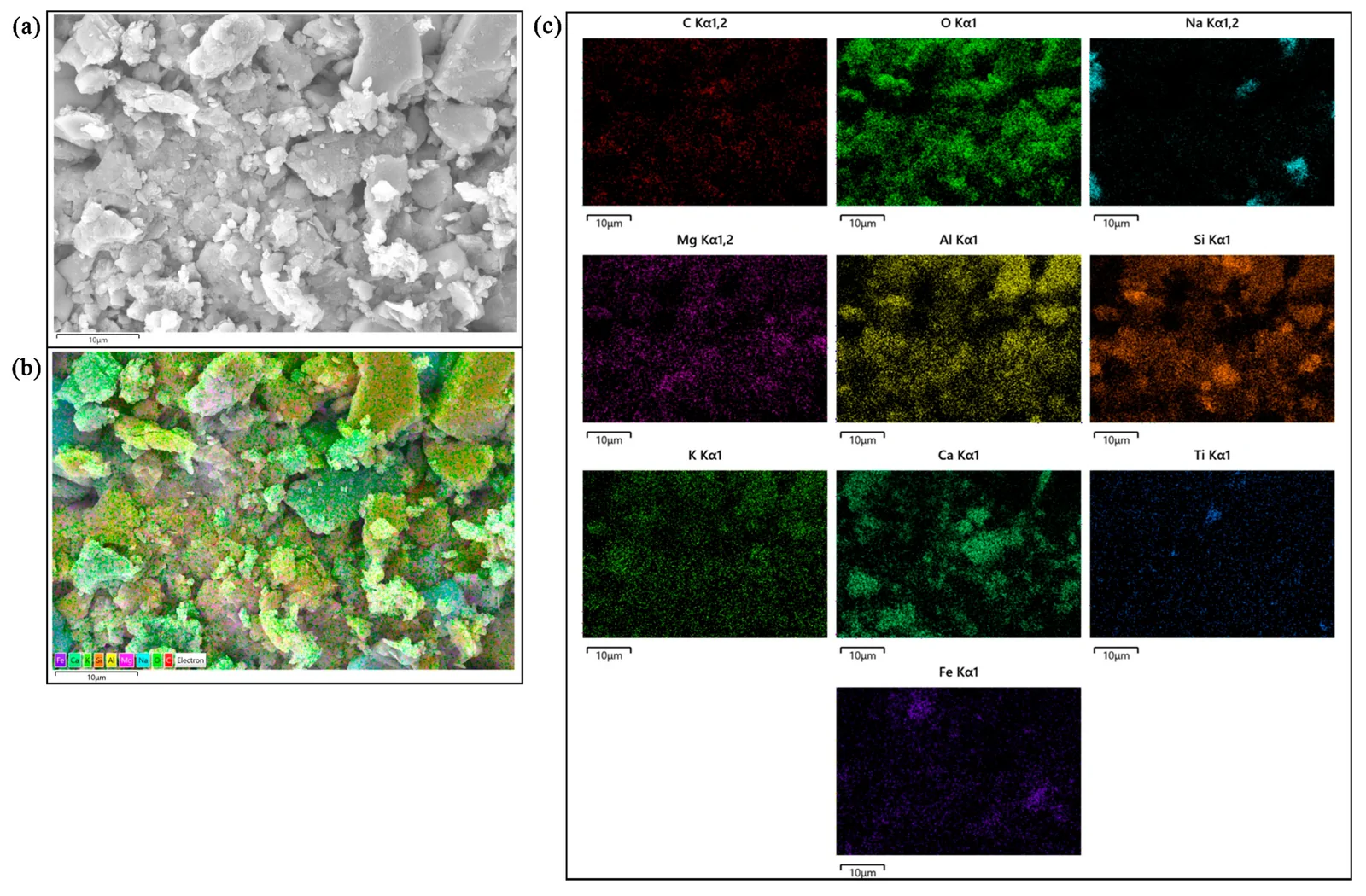
SEM–EDS elemental mapping of Sample C at 5000×. (**a**) Secondary-electron image of the analyzed region. (**b**) Elemental overlay map. (**c**) Individual elemental maps. The corresponding EDS spectrum is provided in Appendix C (Figure A12c).

**Figure 12:**
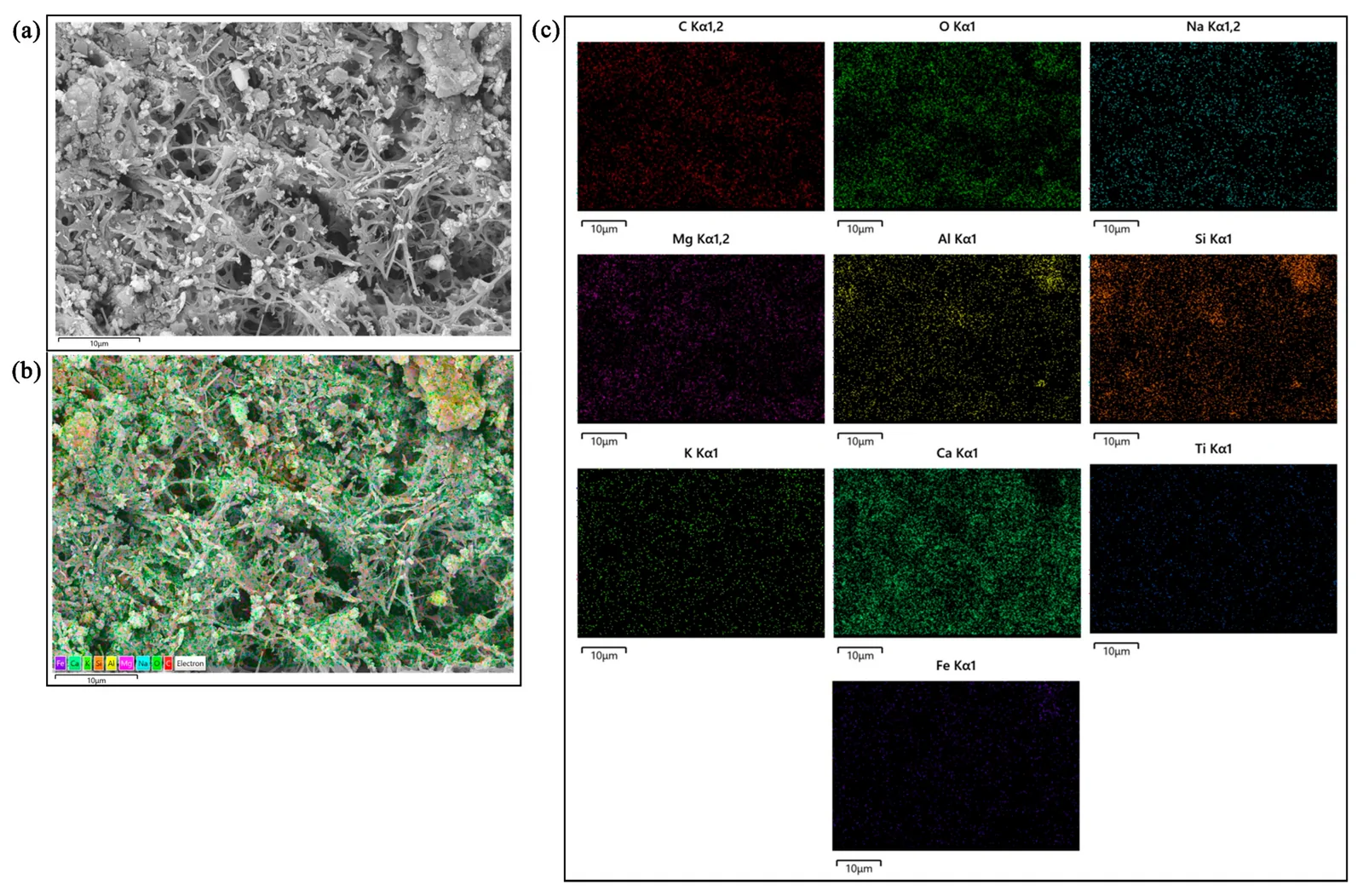
SEM–EDS elemental mapping of Sample D at 5000×. (**a**) Secondary-electron image of the analyzed region. (**b**) Elemental overlay map. (**c**) Individual elemental maps. The corresponding EDS spectrum is provided in Appendix C (Figure A12d).

**Figure 13:**
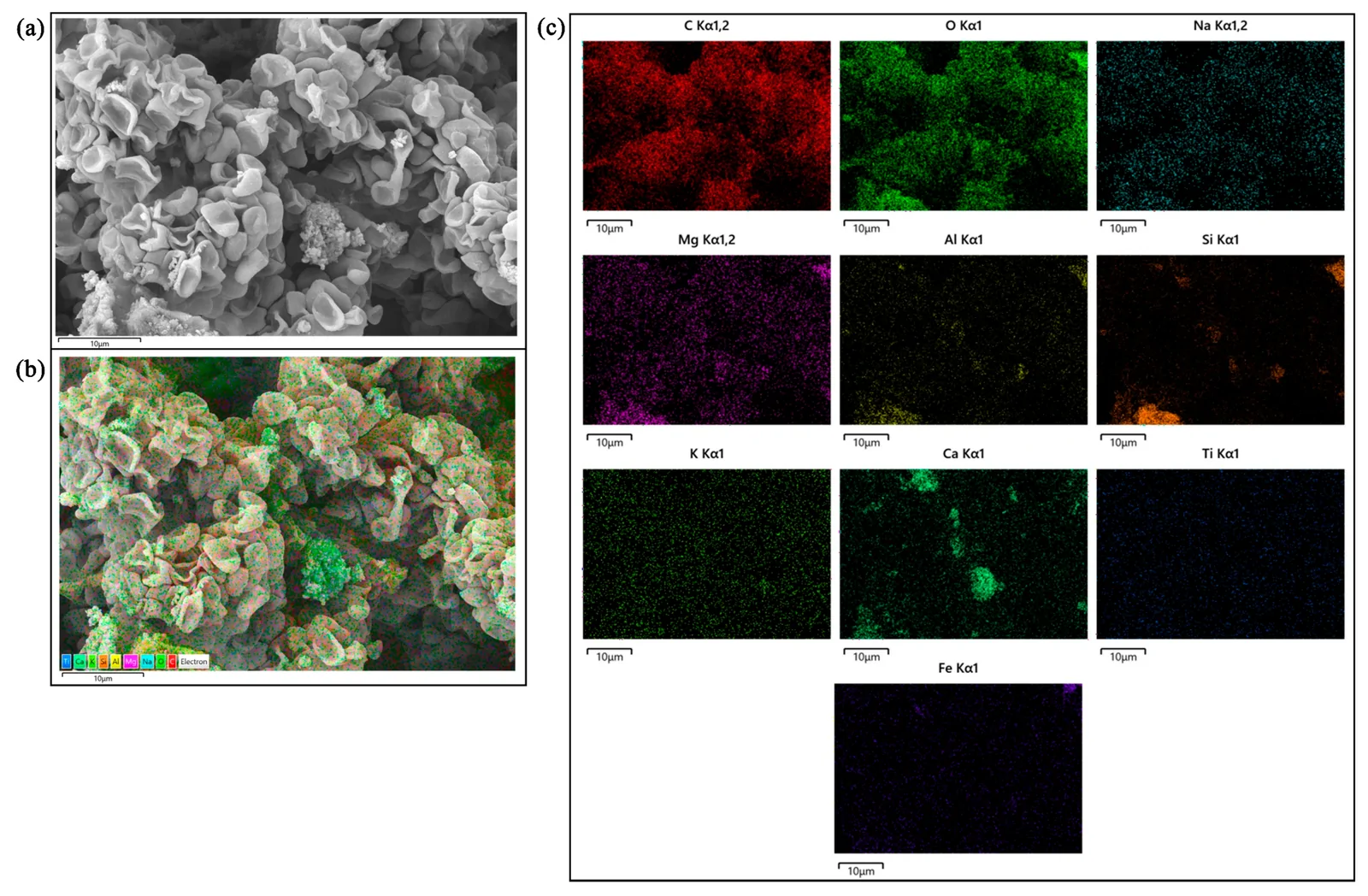
SEM–EDS elemental mapping of Sample E at 5000×. (**a**) Secondary-electron image of the analyzed region. (**b**) Elemental overlay map. (**c**) Individual elemental maps. The corresponding EDS spectrum is provided in Appendix C (Figure A12e).

One EDS-mapped region from each of the Samples A–E is presented at 5000× in Figure 9, Figure 10, Figure 11, Figure 12 and Figure 13. The figures show the corresponding secondary-electron images, elemental overlay maps, and individual element maps.

In the mapped region of A (Figure 9), O and Si are distributed across most particles, while Al and K accompany Si in parts of the field. Ca is localized on some irregular particles, and Na is concentrated in selected particles.

**Figure A12:**
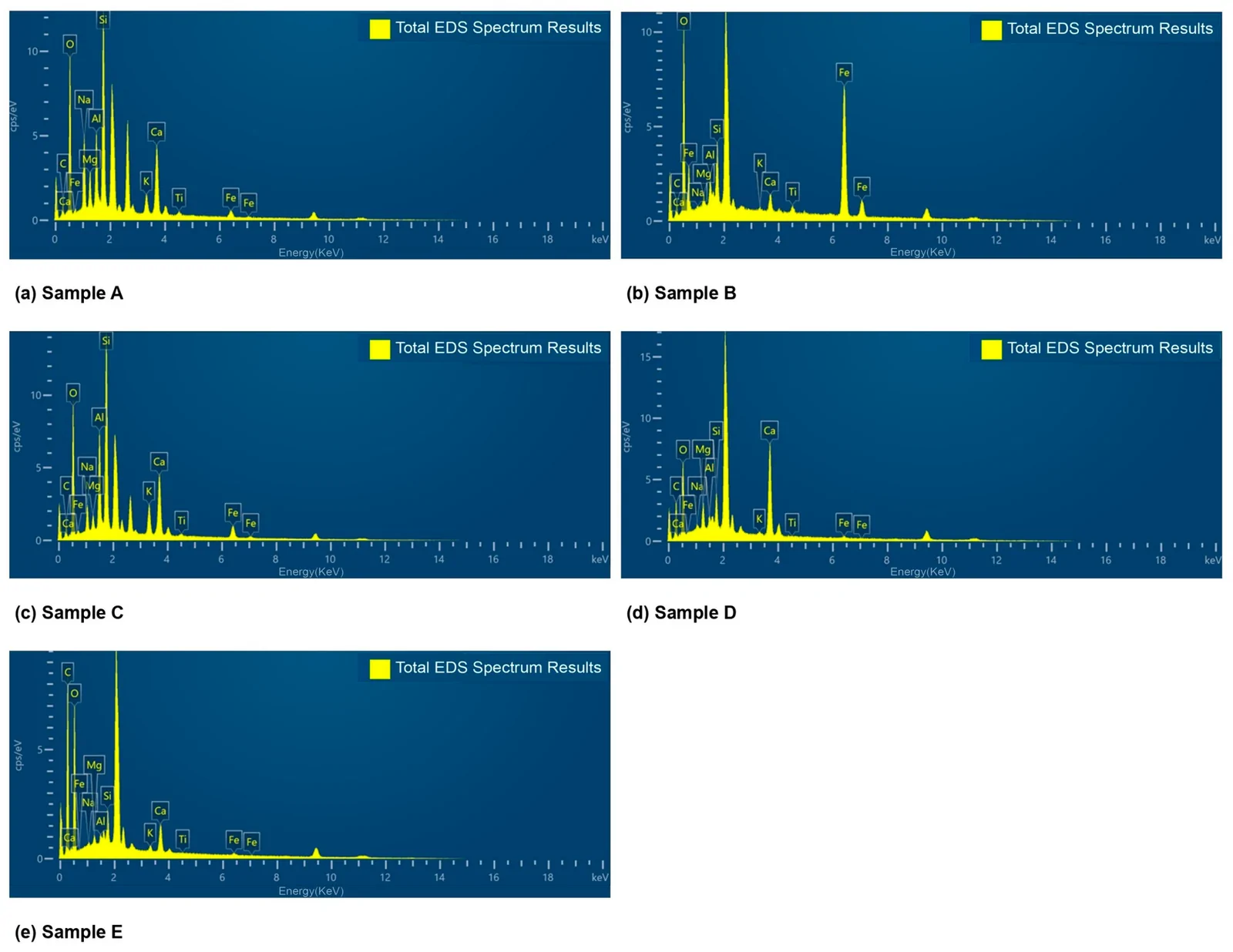
EDS spectra of the mapped regions in Samples A–E.

In the mapped region of B (Figure 10), Fe is widely distributed and partially overlaps with O. The corresponding spectrum shows a prominent Fe signal (Figure A12b, Appendix C), indicating a Fe-rich analyzed region.

In the mapped region of C (Figure 11), O, Si, Al, and K are distributed across particle clusters, and Ca is localized in several areas. Fe is also detected within the field.

The mapped region of D (Figure 12) contains interwoven tubular-fibrous features and granular particles. Ca, O, and C produce prominent signals, while Si and Al signals are weaker.

**Figure 14:**
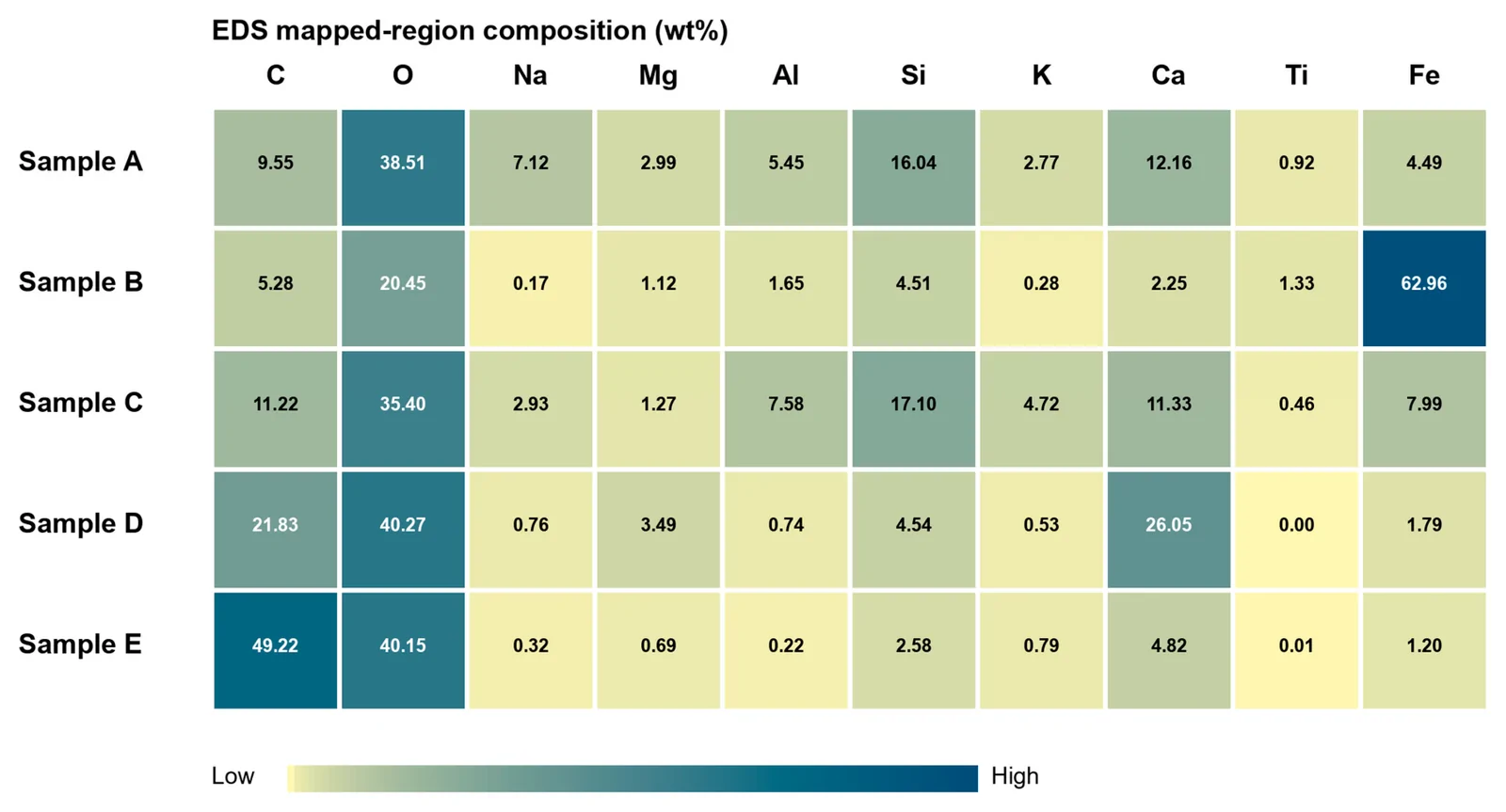
Semi-quantitative EDS composition of the mapped regions in Samples A–E. Values are weight fractions (wt%) and represent local fields of view.

The mapped region of E (Figure 13) contains curled film-like objects and granular particles. C and O are the principal elements reported in the corresponding EDS area-scan output (Figure 14).

The EDS spectra in Appendix C (Figure A12) correspond to the mapped regions in Figure 9, Figure 10, Figure 11, Figure 12 and Figure 13. The principal recorded elements are O, Si, and Ca in A and C, Fe and O in B, Ca, O, and C in D, and C and O in E.

Figure 14 presents the software-reported weight percentages for the five mapped regions. The reported values include 62.96 wt% Fe in the region analyzed in B, 26.05 wt% Ca in D, and 49.22 wt% C in E. The regions analyzed in A and C contain Si at 16.04 and 17.10 wt% and Ca at 12.16 and 11.33 wt%, respectively. Table 4 summarizes the local elemental characteristics, and Appendix C provides the complete numerical outputs.

### 3.4. Mineralogical Composition Determined by XRD

**Figure 15:**
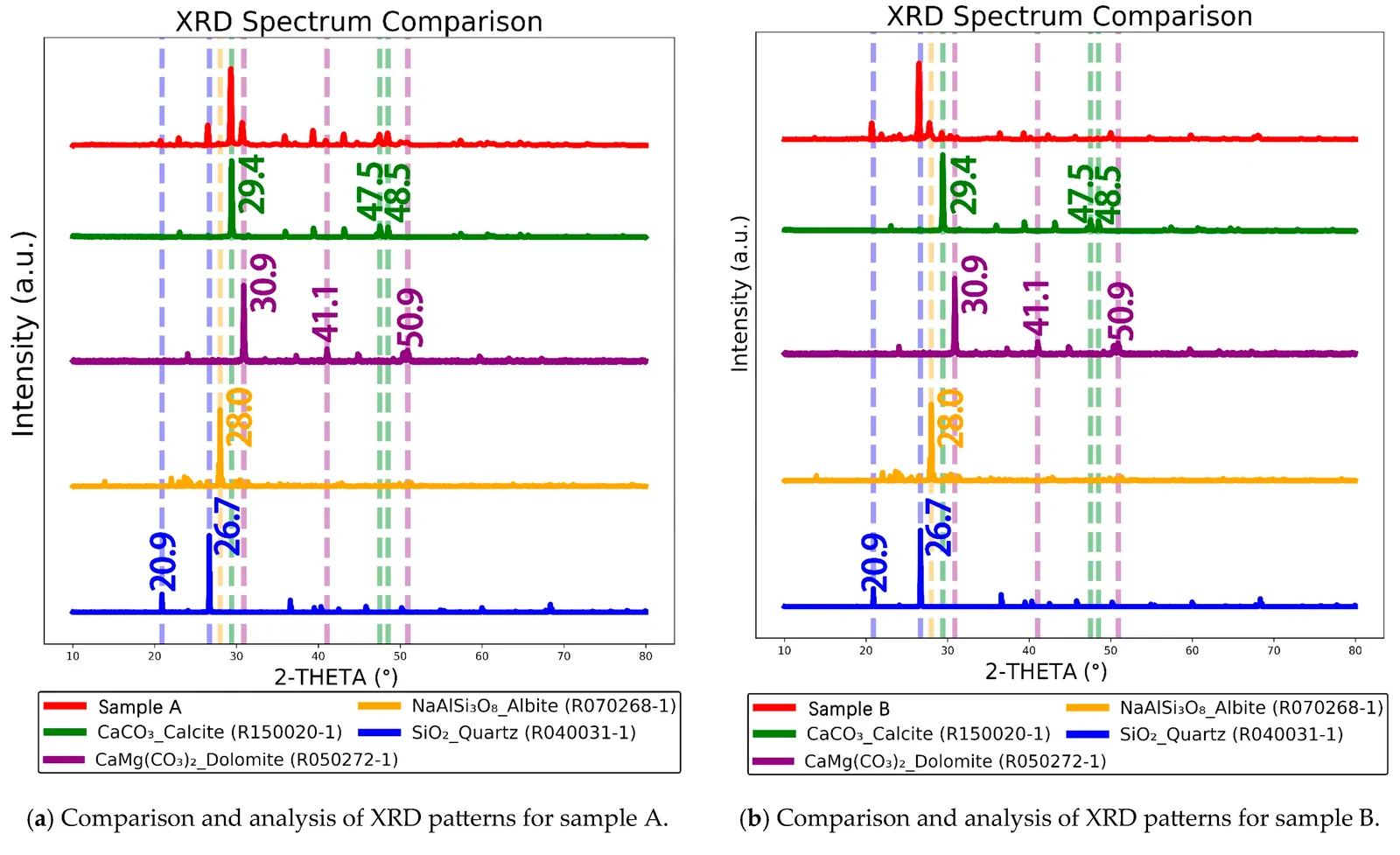

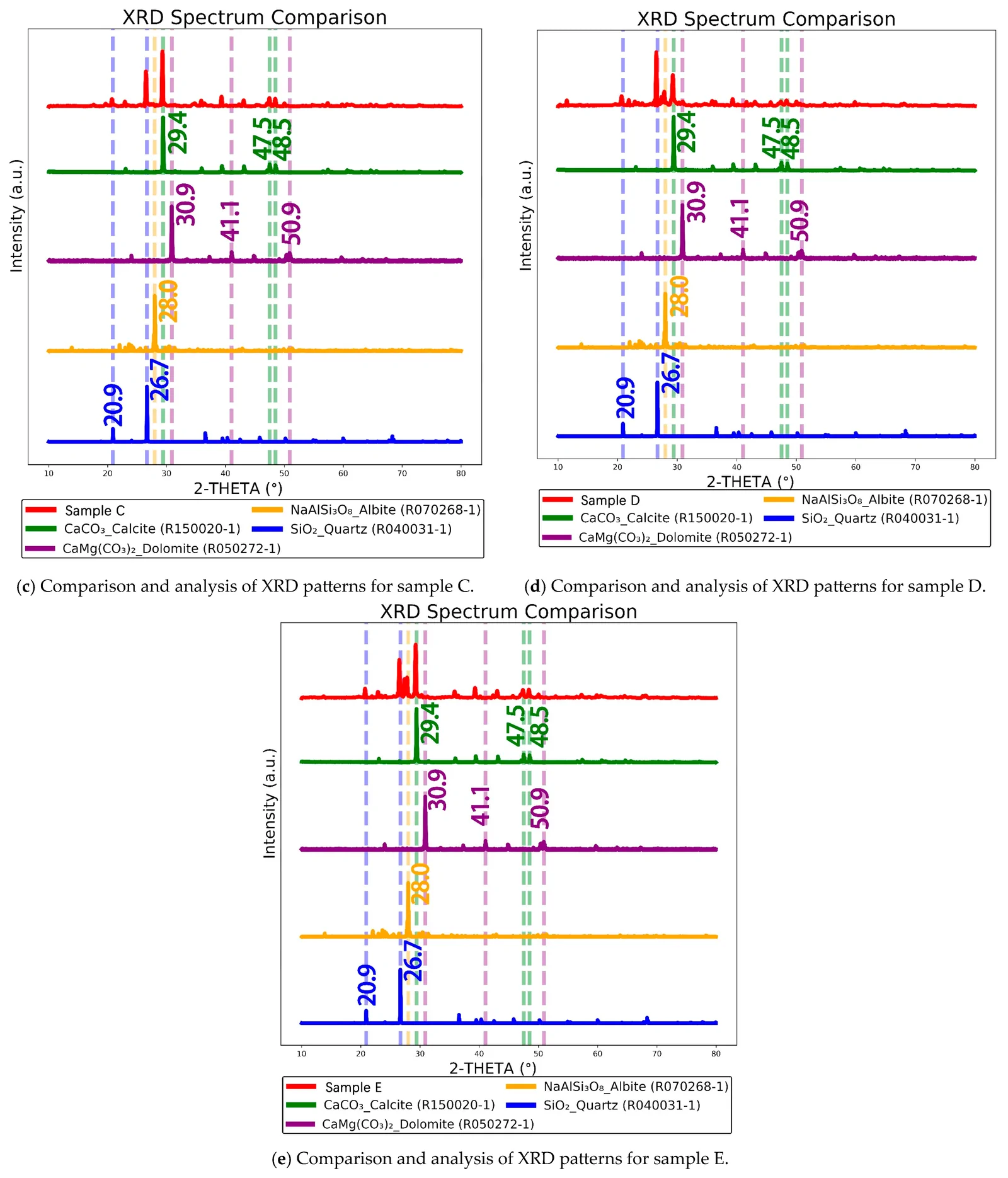
Measured XRD patterns of Samples A–E compared with reference patterns over 10–80° (2θ): (**a**) A; (**b**) B; (**c**) C; (**d**) D; and (**e**) E. The measured sample pattern is shown in red in each panel, with the reference patterns vertically offset below it. Cc and Qz mark reflections assigned to calcite and quartz, respectively, on the measured sample patterns. Assignments to dolomite and albite remain tentative.

Calcite (CaCO_3_) and quartz (SiO_2_) were identified in all five samples (Figure 15). Weak reflections were tentatively matched to dolomite [CaMg(CO_3_)_2_] and albite (NaAlSi_3_O_8_). The highest peak in each measured pattern is assigned to calcite in A, C, and E and to quartz in B and D. The percentages reported below are within-pattern ratios of selected peak heights, not mineral abundances; phase identification remains qualitative.

Sample A has its highest diffraction peak at d ≈ 3.044 Å (2θ = 29.320°), assigned to calcite (Figure 15a). The principal quartz reflection occurs at d ≈ 3.361 Å (2θ = 26.500°). Additional reflections at 2θ = 39.340°, 48.460°, 43.100°, and 35.900° support the identification of calcite.

Sample B has its highest peak at d ≈ 3.361 Å (2θ = 26.500°), assigned to quartz, with a further quartz reflection at d ≈ 4.288 Å (2θ = 20.700°) (Figure 15b).

Sample C has its highest peak at d ≈ 3.044 Å (2θ = 29.320°), assigned to calcite (Figure 15c). The height of the quartz reflection at d ≈ 3.361 Å (2θ = 26.500°) is 63.5% of that of this calcite reflection. Additional reflections at 2θ = 39.340°, 47.460°, 43.100°, and 35.880° are assigned to calcite, while the reflection at 2θ = 20.720° is assigned to quartz.

Sample D has its highest peak at d ≈ 3.361 Å (2θ = 26.500°), assigned to quartz (Figure 15d). The height of the calcite reflection at d ≈ 3.048 Å (2θ = 29.280°) is 57.6% of that of this quartz reflection. The quartz reflection at 2θ = 20.660° and calcite reflections near 2θ = 39.320° and 48.360° further support the identification of both phases.

Sample E has its highest peak at d ≈ 3.050 Å (2θ = 29.260°), assigned to calcite (Figure 15e). The height of the quartz reflection at d ≈ 3.361 Å (2θ = 26.500°) is 71.7% of that of this calcite reflection. The weak reflection at d ≈ 3.880 Å (2θ = 22.900°) is tentatively associated with feldspar.

## 4. Discussion

### 4.1. Material Heterogeneity in the Context of the Documented Repair History

The repeated repairs described in Section 2.1 provide a historical context for the material variation observed at the East Gate. Samples B, D, and E were collected from the inner wall of the barbican, yet B and E contain abundant coarse grains, whereas D-1 and D-2 are dominated by fine-grained matrices. Material differences therefore occur within the same architectural component as well as between components. Different materials introduced during successive repairs may have contributed to these contrasts, but the sampled fragments cannot be assigned to specific construction or repair phases from the present evidence. These local contrasts support recording individual masonry units and their interfaces in conservation surveys.

### 4.2. Integrated Interpretation of Mineral Assemblages and Petrographic Textures

The five fragments share the confirmed XRD phases calcite and quartz, but their petrographic textures differ. Samples A and C have fine-grained continuous matrices, B and E contain abundant coarse grains, and the two sections of Sample D are matrix-dominated but optically distinct. The observed heterogeneity therefore concerns matrix–grain organization and local elemental distributions, rather than simply the presence or absence of calcite and quartz.

Related studies provide two relevant contexts for this comparison. Li et al. reported quartz, feldspar, and a minor amount of calcite in historical bricks from the Xi’an city wall [21]. Studies of historical mortars emphasize the relationships between binders, aggregates, and reaction boundaries [17]. Wei et al. distinguished aerial lime mortars from city-wall joints and a pozzolanic mortar from a tomb in Anhui [26]. These comparisons make both mineral occurrence and matrix–grain texture relevant to the interpretation of the East Gate fragments. In this sample set, grain size and matrix continuity provide the clearest textural distinctions between materials.

XRD provides the phase-specific evidence for calcite and quartz. Petrography describes matrix–grain relationships, while the recorded optical characteristics do not uniquely identify these phases. The Si–O associations in A and C are compatible with silicate-bearing constituents, and the prominent Ca, O, and C signals in D are compatible with carbonate-bearing constituents. These local elemental associations are consistent with, but do not independently confirm, the quartz and calcite identified by XRD. Dolomite and albite remain tentative assignments without independent petrographic or EDS confirmation. In B, the Fe-rich EDS region indicates local chemical variation within a fragment whose bulk XRD pattern contains quartz, rather than the composition of the entire fragment.

### 4.3. Moisture Exposure and Possible Material Alteration

Sample C was collected from the gate culvert and contains fine fissures and grain clusters in the thin section. Its XRD pattern includes calcite and quartz. Studies of weathered historic bricks show that environmental exposure can alter surface morphology and mineral assemblages [27,28]. Moisture-related alteration is therefore a possible explanation to examine at this location. The spatial relationship between fissures, carbonate-bearing constituents, and moisture pathways is relevant to subsequent conservation surveys.

### 4.4. Filamentous and Film-like Features in Samples D and E

**Figure 16:**
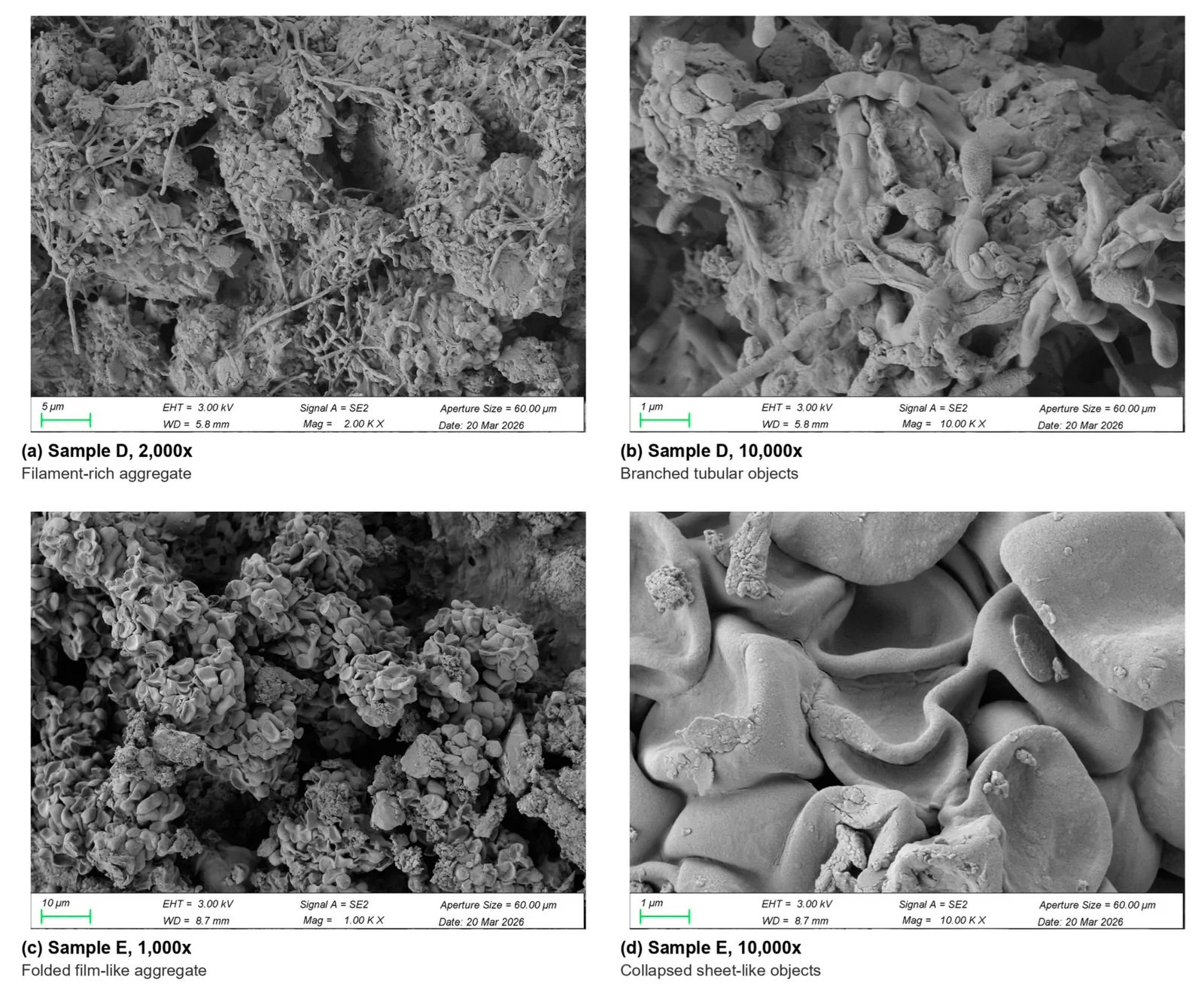
Representative SEM morphologies in Samples D and E, showing interwoven filamentous and tubular features in D and folded film-like features in E.

SEM images of Sample D show filamentous and tubular features extending between granular aggregates, with some features crossing or overlapping (Figure 16). Local branching, curvature, and adhering fine particles are visible. Based on the image scale bars, the fine filaments are predominantly submicrometer in size, while the thicker tubular objects measure approximately 1–3 μm. Other SEM images show short rod-like features. Sample E contains thin, sheet-like objects with smooth surfaces and curled edges, including folded and cup-like forms. Individual sheets range from a few micrometers to approximately 10 μm and occur among granular particles. Table 5 summarizes these morphologies and the elemental features of the mapped regions.

**Figure 17:**
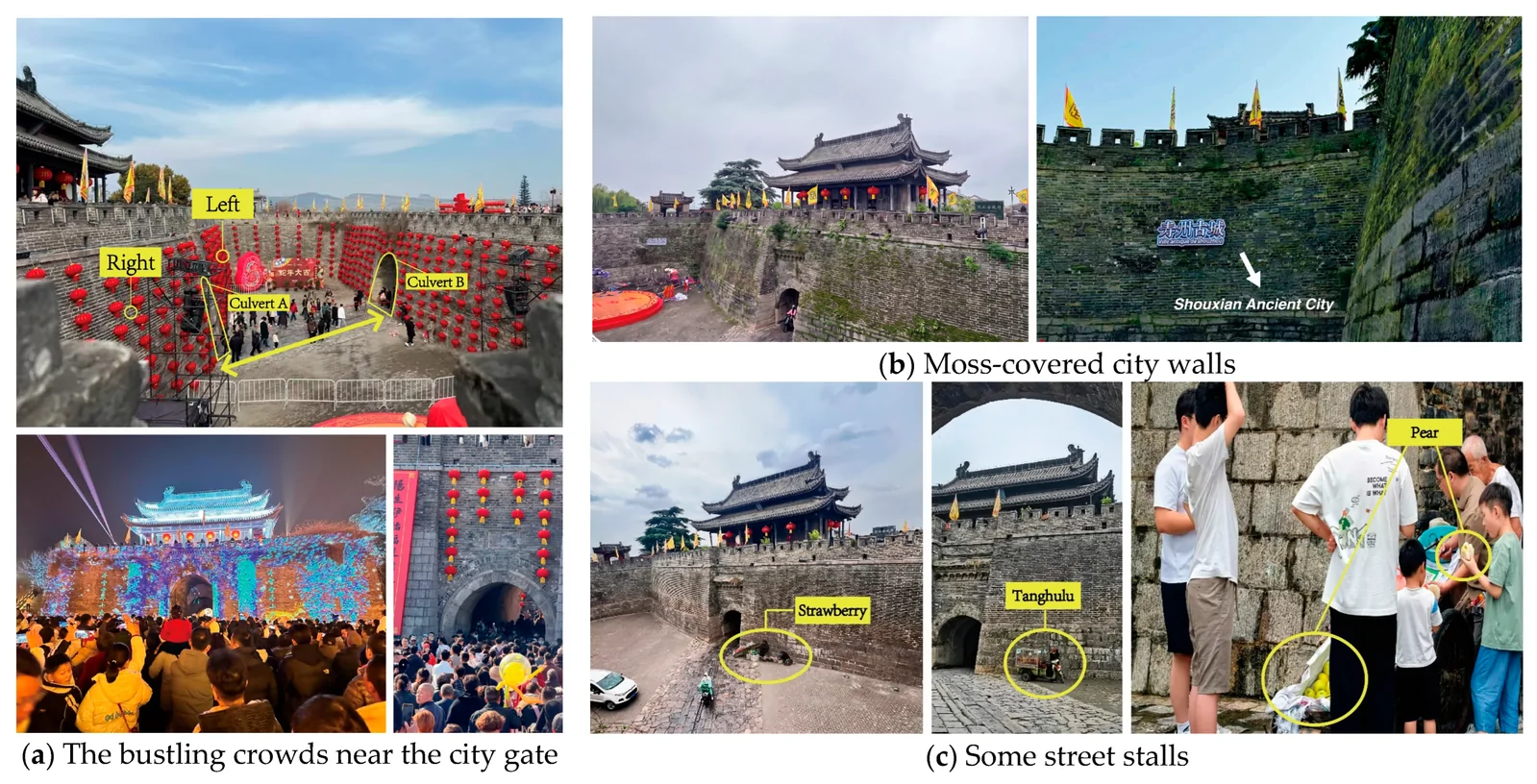
On-site investigation. The Chinese characters for “寿州古城” shown in the picture are indeed the name of this ancient city. (Image source: Photo by the author).

The site survey recorded pedestrian activity, street stalls, and moss and herbaceous growth near the East Gate and its barbican (Figure 17). Street stalls, including fruit vendors, were observed close to the sampling locations for D and E.

Zhao et al. reported microbial communities on the Shouxian city wall, including Actinobacteria, Cyanobacteria, and Ascomycota fungi, and discussed hyphal-like coverings and particle retention by extracellular polymers [29]. Biological attachment and environmental residues are possible explanations for the filamentous features in D and the film-like features in E. Their origin remains unresolved, and the present images and area-scan EDS data do not establish whether they are biological material, mineral alteration products, or other residues. Because the EDS results describe mixed mapped regions, the reported Ca and C signals cannot be assigned specifically to individual filaments or films or used to link these features to calcite, quartz, or another mineral phase.

### 4.5. Calcite-Bearing Constituents in the Sampled Masonry Materials

Calcite occurs together with quartz in the analyzed fragments. Its strongest reflection exceeds the quartz reflection in A, C, and E, whereas quartz produces the strongest reflection in B and D. In the masonry context, calcite may derive from carbonate aggregates, lime-bearing materials, or secondary deposition. These possible sources give different meanings to the same phase assemblage and make the position of carbonate within the matrix, grains, and masonry interfaces important for interpreting material use.

### 4.6. Conservation Implications and Study Limitations

The contrast between fine-grained matrix-dominated materials and coarse-grained materials highlights grain size, matrix continuity, and mineral assemblages as useful descriptors for conservation surveys. Recording these features for individual masonry units and their interfaces would allow material differences to be mapped across the East Gate and its barbican. The culvert and wall base are also relevant locations for moisture and soluble-salt investigations. Subsequent repair-material selection should combine this characterization with chemical, physical, mechanical, and durability testing.

The study covers five near-surface fragments, with their individual construction dates and the wider distribution of material types remaining unresolved. Powder preparation alters the original particle arrangement, while irregular particle geometry limits quantitative EDS interpretation. The recorded carbon signal may include a contribution from the conductive adhesive. Grain-specific compositions, the original pore network, and the origins of the filamentous and film-like features remain unresolved. Bulk chemical compositions and physical and durability properties were not measured. Spatially replicated sampling, polished-section SEM–EDS with targeted mineral analyses, and ICP-based bulk chemical analysis would extend the textural and chemical comparison. Targeted spectroscopic and thermal analyses would help characterize the unusual features, while physical and durability testing would support the evaluation of repair materials.

## 5. Conclusions

This study fills a gap in the reference evidence for petrographic and mineral data of historical masonry of ancient city walls in the Jianghuai (between the Yangtze and Huaihe rivers) transition zone of China. Analysis of the five sampled fragments leads to three conclusions.

(1) Shared mineral phases do not imply uniform masonry materials. Although calcite and quartz occur in all five fragments, matrix–grain textures differ between fine-grained matrix-dominated materials and materials containing abundant coarse grains. Together with the contrast between D-1 and D-2 and the local elemental differences, this demonstrates heterogeneity both between sampled fragments and within an individual fragment.

(2) These differences are compatible with variation in materials used during construction or repair, but they do not identify specific historical phases or their chronology. The mineralogical results also do not establish the origin of the filamentous and film-like features, which remains unresolved.

(3) Conservation documentation should record individual masonry units and material interfaces, using grain size, matrix continuity, and mineral assemblages to distinguish areas requiring further assessment. Such documentation can guide targeted investigations of contrasting materials. Repair-material selection should additionally consider physical, mechanical, and durability properties.

## Figures and Tables

**Table 1:** Sampling locations and surface appearances of Samples A–E.

Sample Number	Sampling Locations	Surface Appearance
A	Lower half of the front wall facing the city from the East Gate	Black
B	Inner wall of the East Gate barbican	Red
C	Inner wall of the East Gate culvert	Yellow
D	Inner wall of the East Gate barbican	Gray and whitish areas
E	Inner wall of the East Gate barbican	Miscellaneous colors

Source: Author statistics.

**Table 2:** Experimental procedure, main parameters and applications.

Methods	Samples and Sample Preparation	Instruments/Conditions	Output Results	Applications for This Article
Polarizing microscope	A, B, C, D-1, D-2, E; thin sections approximately 30 μm thick	Room temperature; Olympus GX71 (Olympus, Tokyo, Japan); PPL/XPL paired; 500, 200, 100, 50 μm scales	222 images	To interpret matrix, aggregate, sorting, fracture, and optical characteristics.
XRD	A–E; ground powder	SmartLab SE (Rigaku, Akishima, Japan); Cu Kα; 1.54060 Å; 10–80°; 2°/min	Diffractograms and phase-identification records	Qualitative identification of mineral phases.
SEM	A–E; powder bonded with conductive adhesive and sputtered with gold	Sigma 360 FE-SEM (ZEISS, Oberkochen, Germany); SE; 3.0 kV; 50–10,000×; WD 5.5–8.9 mm	70 multi-magnification topography images	To compare powder particle aggregation patterns and local surface morphology.
EDS	A–E; same as SEM	15.0 kV; 5000×; WD 6.7–7.9 mm; C, O, Na, Mg, Al, Si, K, Ca, Ti, Fe	Elemental surface distribution, energy dispersive spectroscopy, and semi-quantitative numerical values	To describe local elemental distributions within each mapped region.

Source: Author statistics.

**Table 3:** Matrix–grain textures observed in the six thin sections.

Sample	Matrix Characteristics	Grain and Fracture Characteristics
A	Fine-grained continuous matrix	Dispersed fine, sub-angular grains and localized grain clusters; coarse grains are scarce.
B	Discontinuous dark matrix	Abundant sub-angular to angular grains, commonly 100–300 μm and locally >500 μm; poor sorting.
C	Fine-grained continuous matrix	Fine grains with localized grain clusters and linear fissures.
D-1	Fine-grained continuous matrix	Few coarse grains, small dark spots, and narrow fractures.
D-2	Continuous translucent matrix	Sparse grain clusters and a few dark spots.
E	Dark intergranular matrix	Abundant sub-angular to subrounded grains approximately 200–500 μm in size, including composite particles.

Source: Author statistics.

**Table 4:** Principal elemental features of the EDS-mapped regions in Samples A–E.

Sample	Local Elemental Characteristics
A	O and Si are widespread, with localized Ca signals.
B	Fe is widespread and partially overlaps with O.
C	O, Si, Al, and K occur across particle clusters, with localized Ca.
D	Ca, O, and C are prominent within the mapped particle assemblage.
E	C and O are prominent in the analyzed area containing folded film-like features.

Source: Author statistics.

**Table 5:** SEM observations and local EDS results for Samples D and E. (EDS values refer to the mapped regions shown in Figure 12 and Figure 13).

Sample	Key Morphologies	Elemental Features of the Mapped Region
D	Interwoven fine filaments, branched tubular structures, and short rod-like objects; surfaces with adhering fine particles	Prominent Ca, O, and C signals
E	Smooth, thin flakes that curl, collapse, and aggregate into cup-like or membrane-like forms	Prominent C and O signals

**Figure A1:**
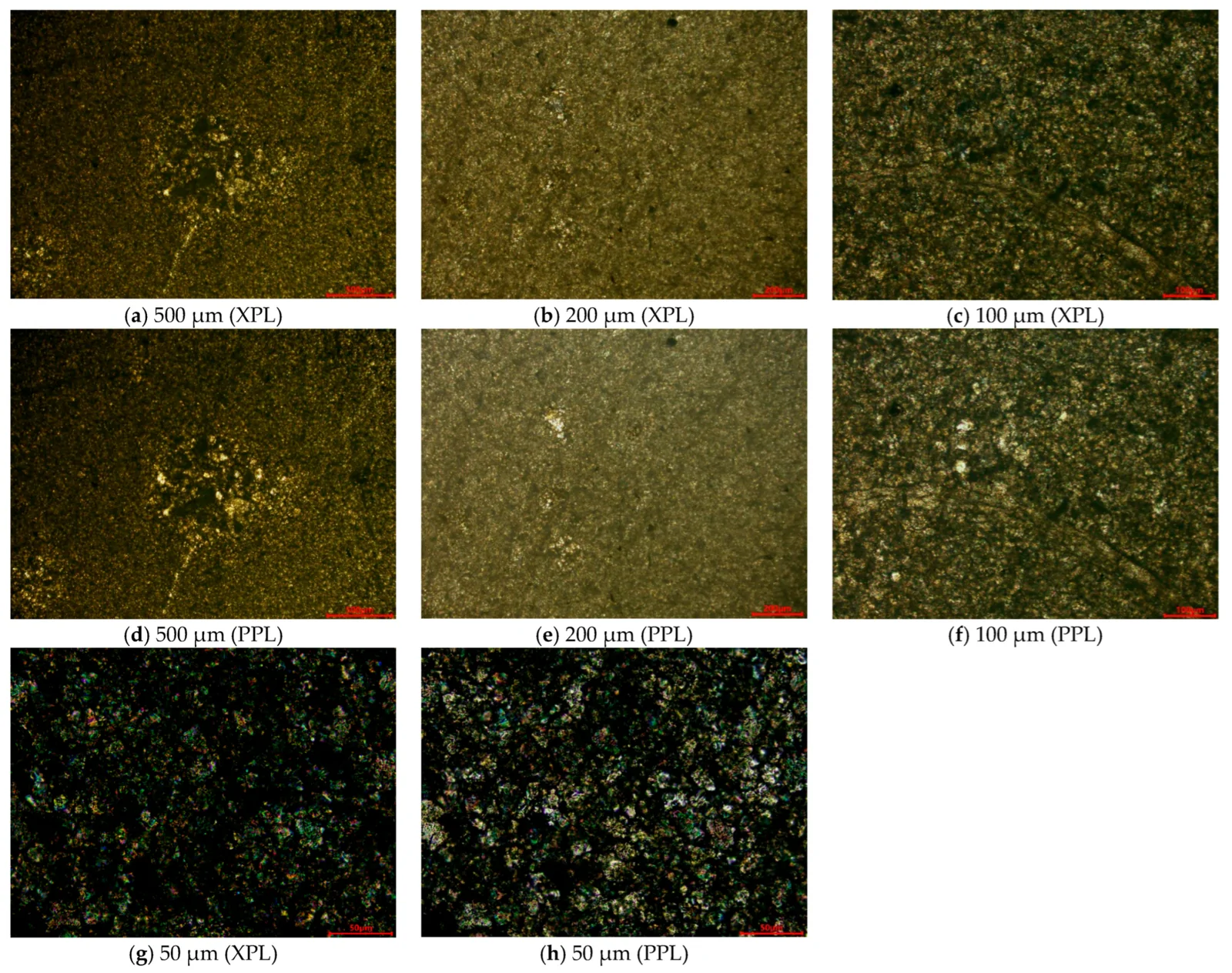
Multi-scale XPL and PPL photomicrographs of Sample A. Scale bars are embedded in each panel.

**Figure A2:**
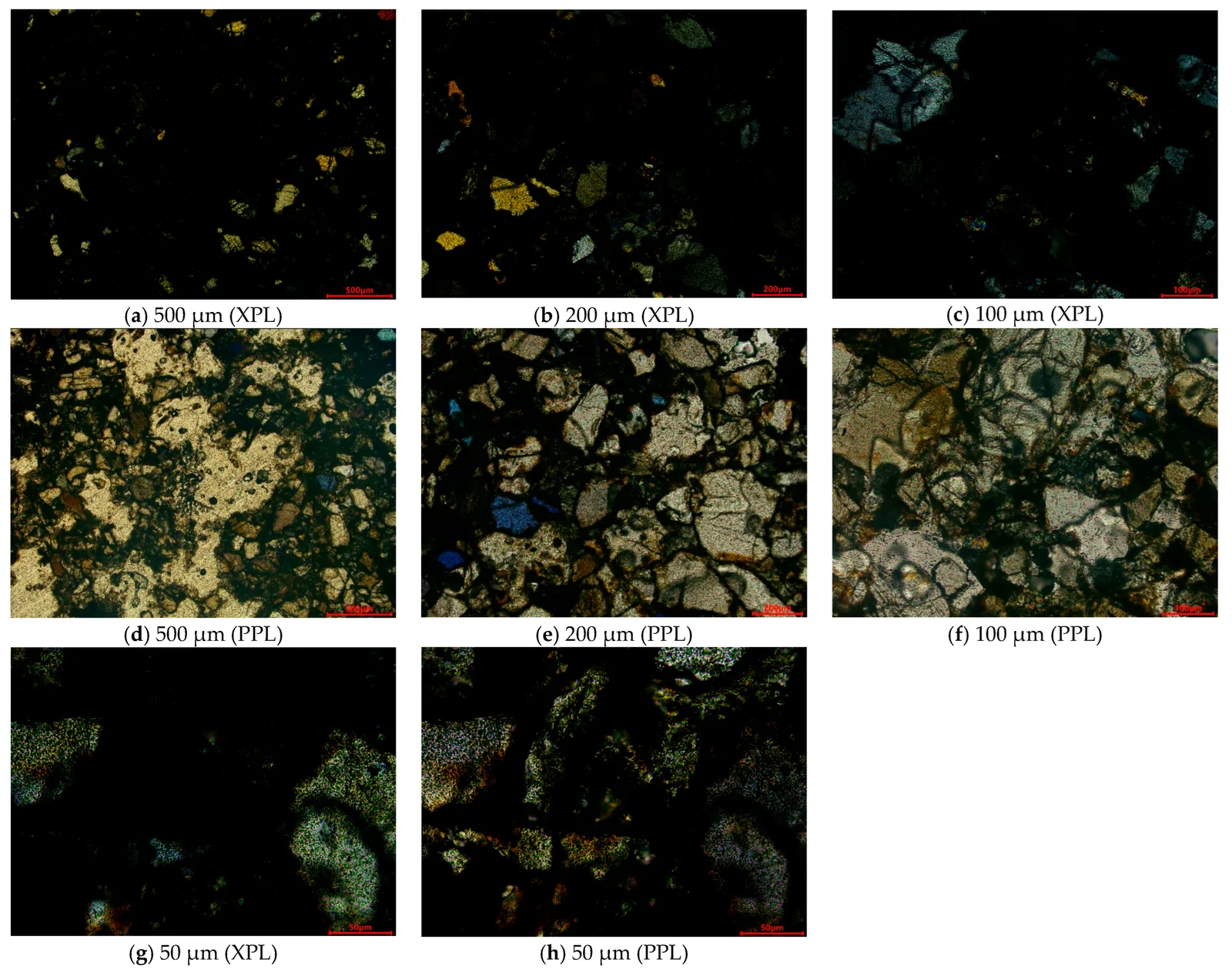
Multi-scale XPL and PPL photomicrographs of Sample B. Scale bars are embedded in each panel.

**Figure A3:**
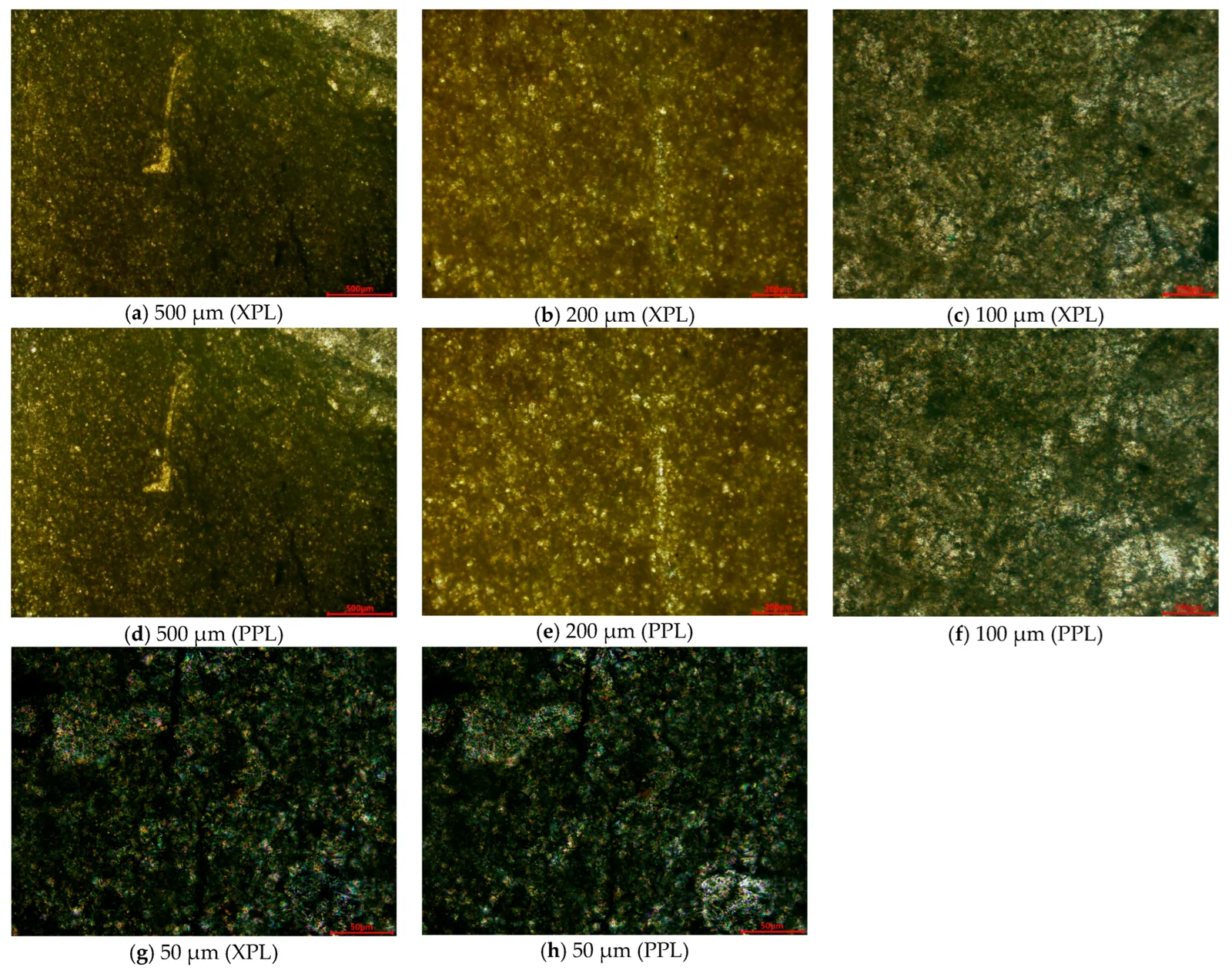
Multi-scale XPL and PPL photomicrographs of Sample C. Scale bars are embedded in each panel.

**Figure A4:**
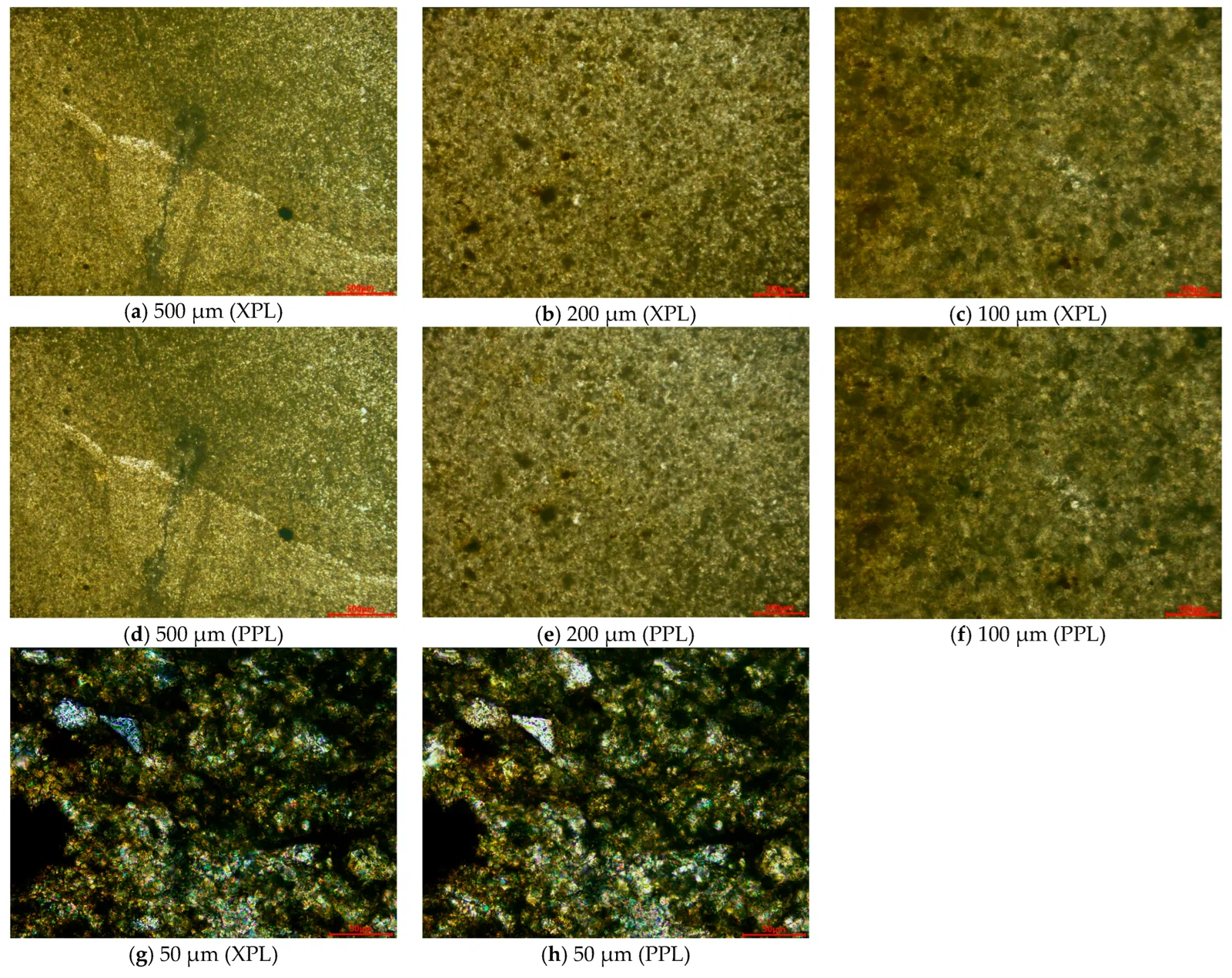
Multi-scale XPL and PPL photomicrographs of Sample D-1. Scale bars are embedded in each panel.

**Figure A5:**
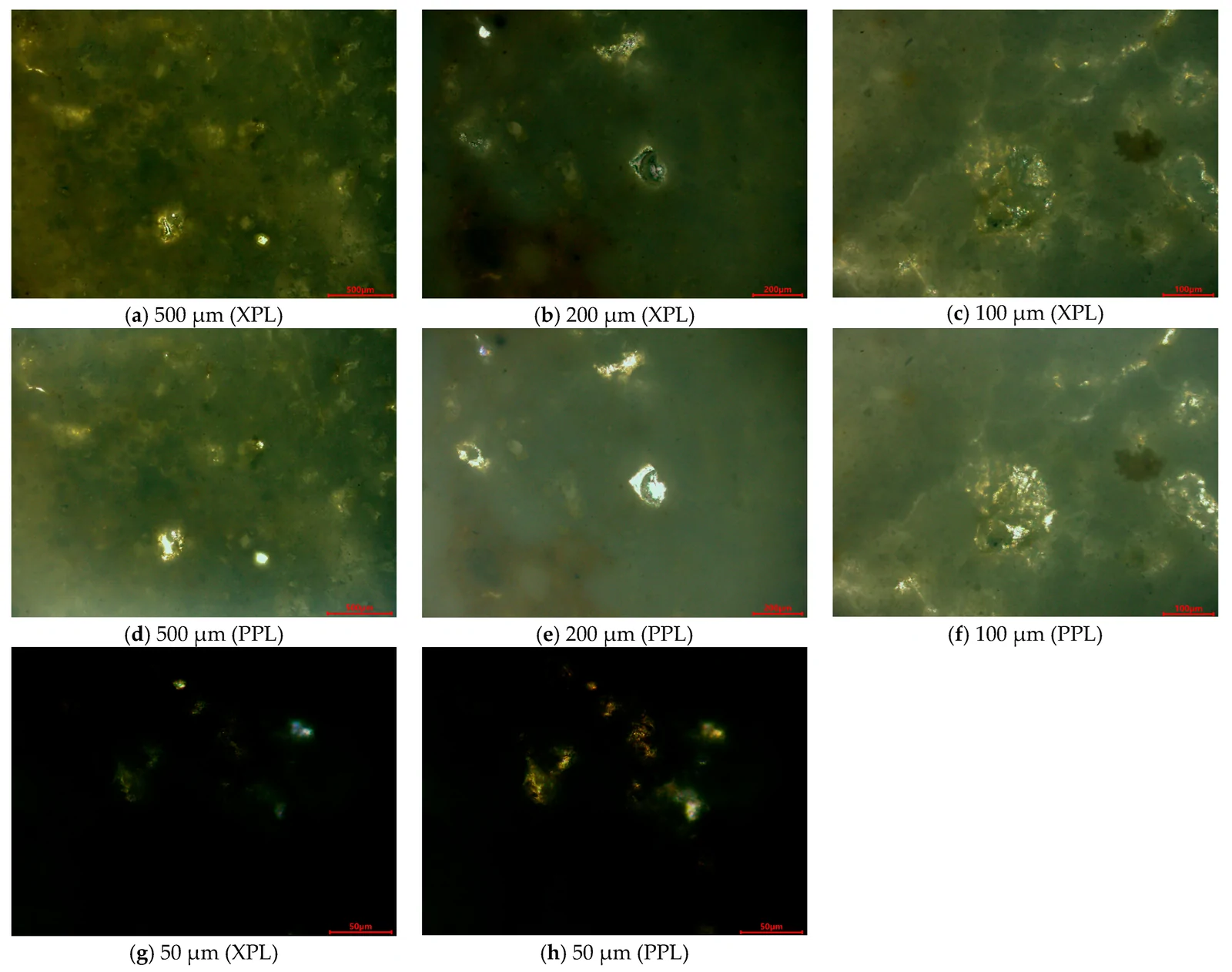
Multi-scale XPL and PPL photomicrographs of Sample D-2. Scale bars are embedded in each panel.

**Figure A6:**
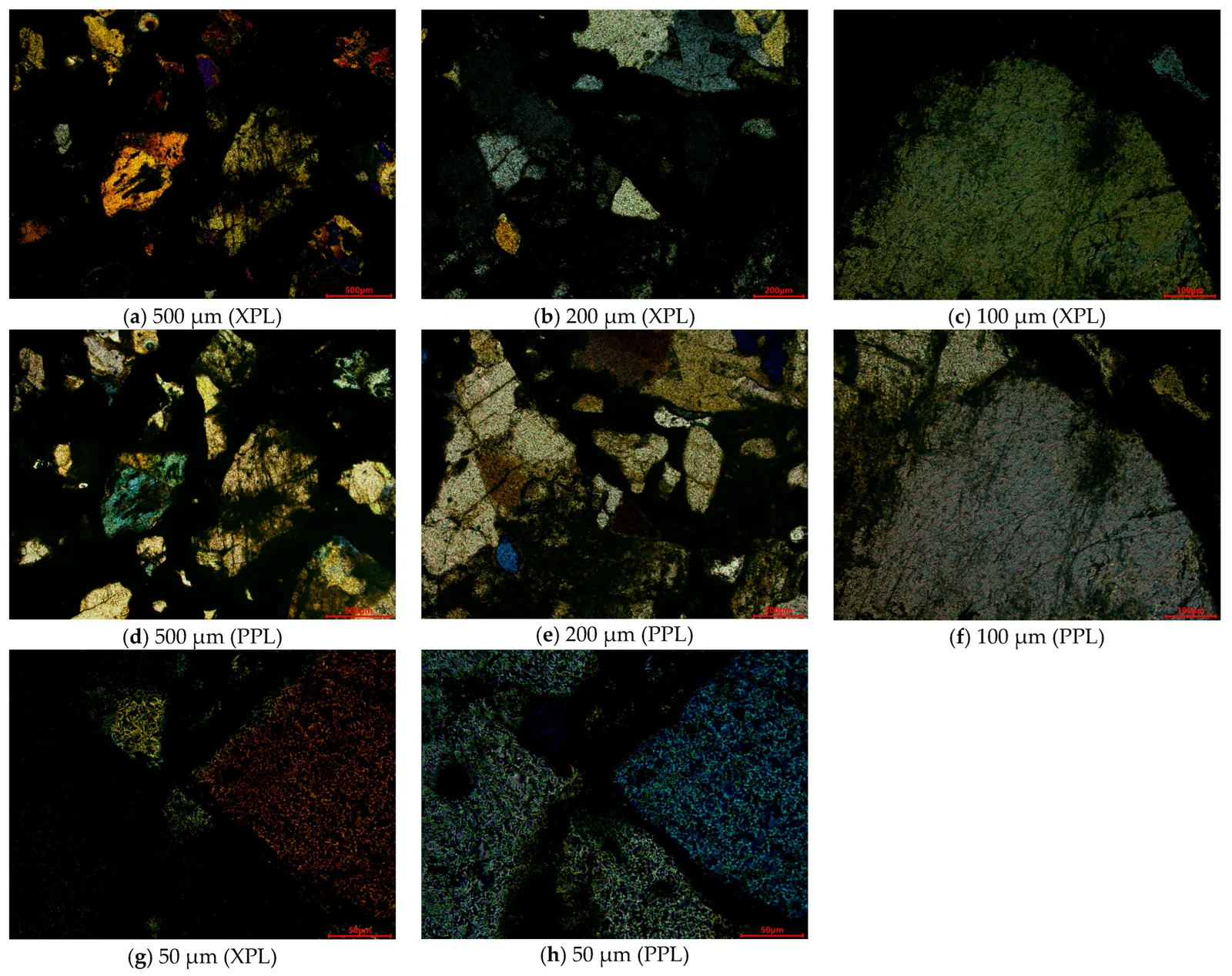
Multi-scale XPL and PPL photomicrographs of Sample E. Scale bars are embedded in each panel.

**Figure A7:**
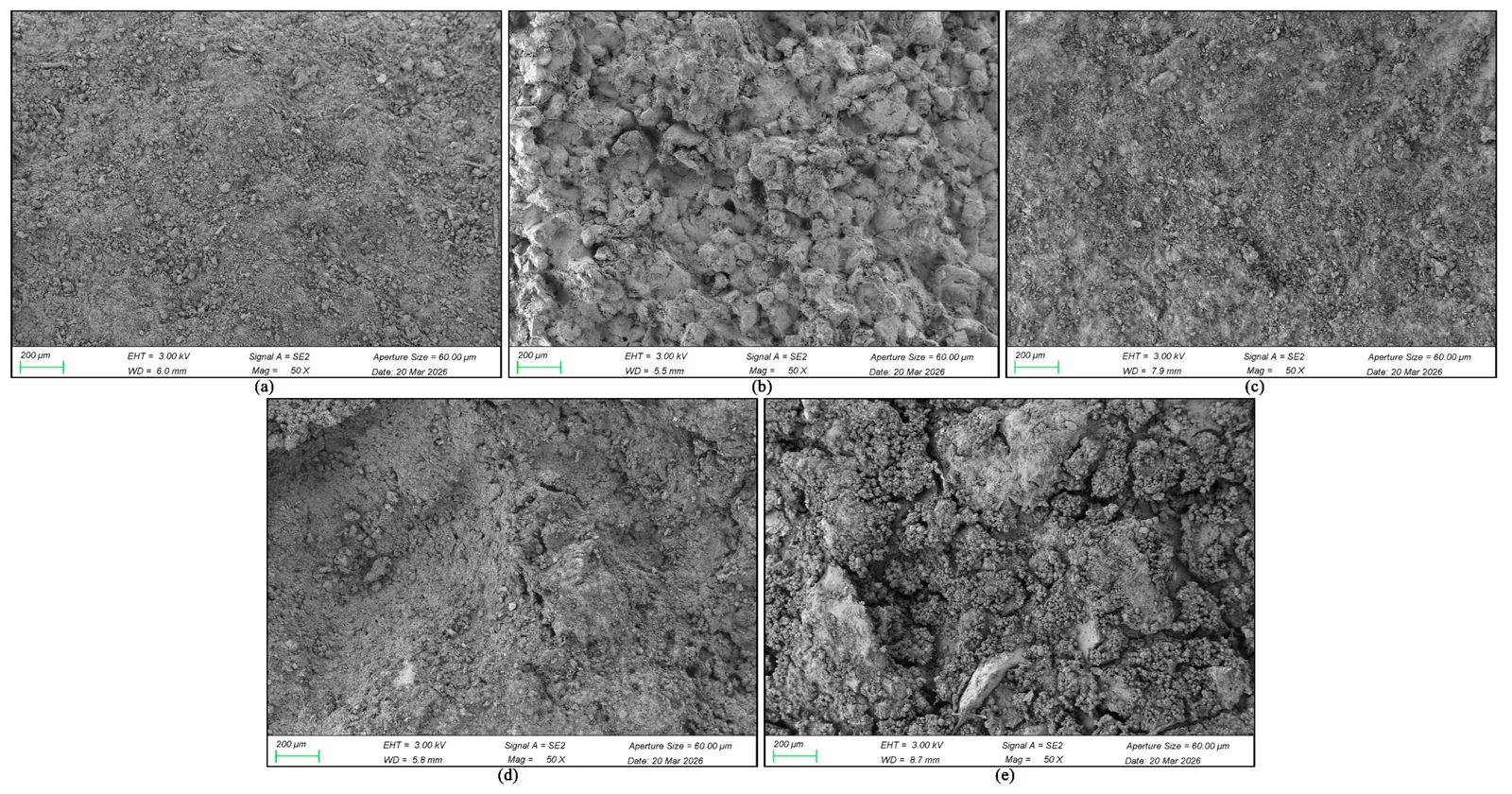
SEM secondary-electron images of Samples A-E at 50×. (**a**) SEM morphology of particles in sample A; (**b**) SEM morphology of particles in sample B; (**c**) SEM morphology of particles in sample C; (**d**) SEM morphology of particles in sample D; (**e**) SEM morphology of particles in sample E. (Image source: compiled by the author based on SEM analysis results).

**Figure A8:**
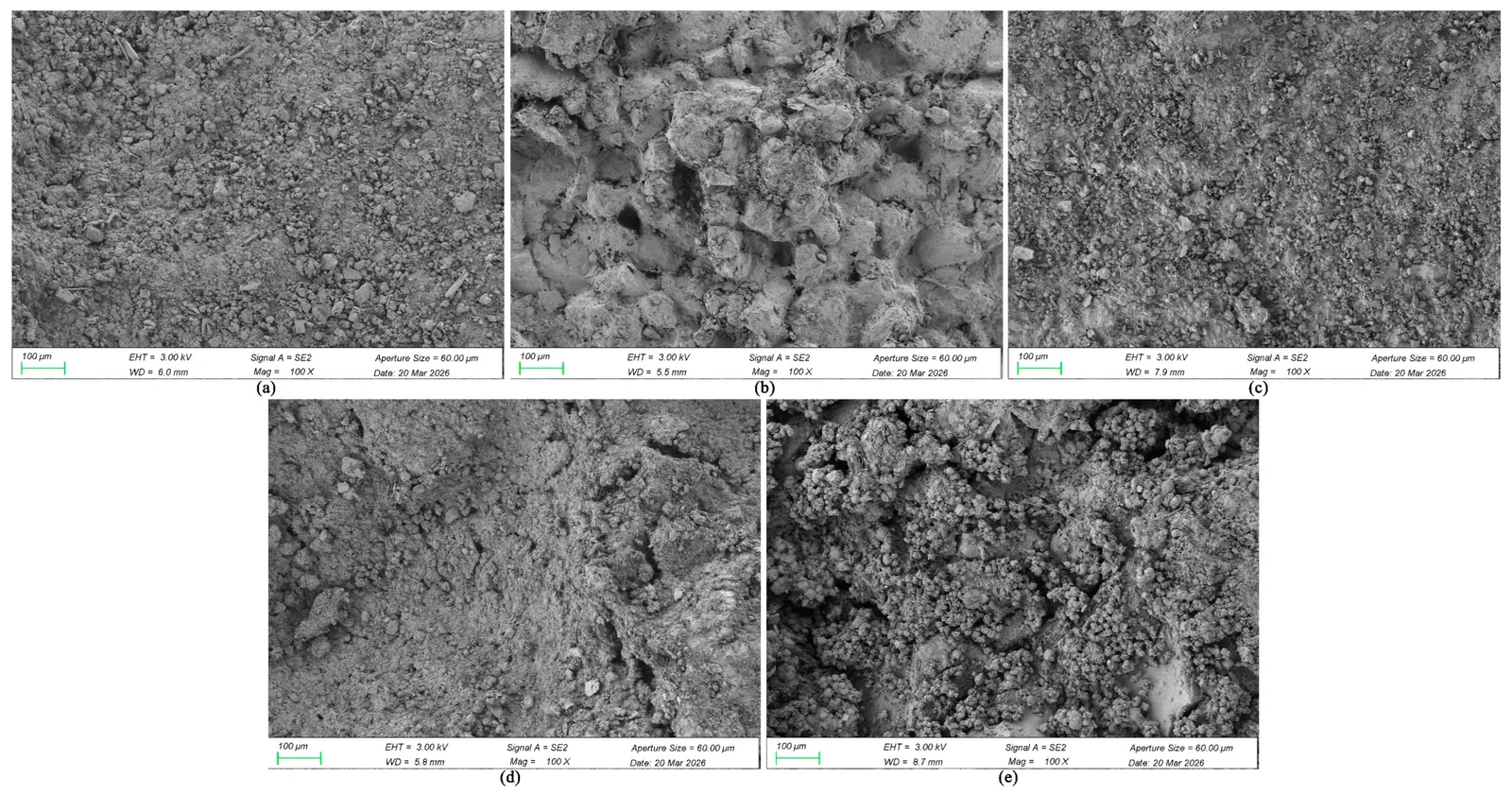
SEM secondary-electron images of Samples A-E at 100×. (**a**) SEM morphology of particles in sample A; (**b**) SEM morphology of particles in sample B; (**c**) SEM morphology of particles in sample C; (**d**) SEM morphology of particles in sample D; (**e**) SEM morphology of particles in sample E. (Image source: compiled by the author based on SEM analysis results).

**Figure A9:**
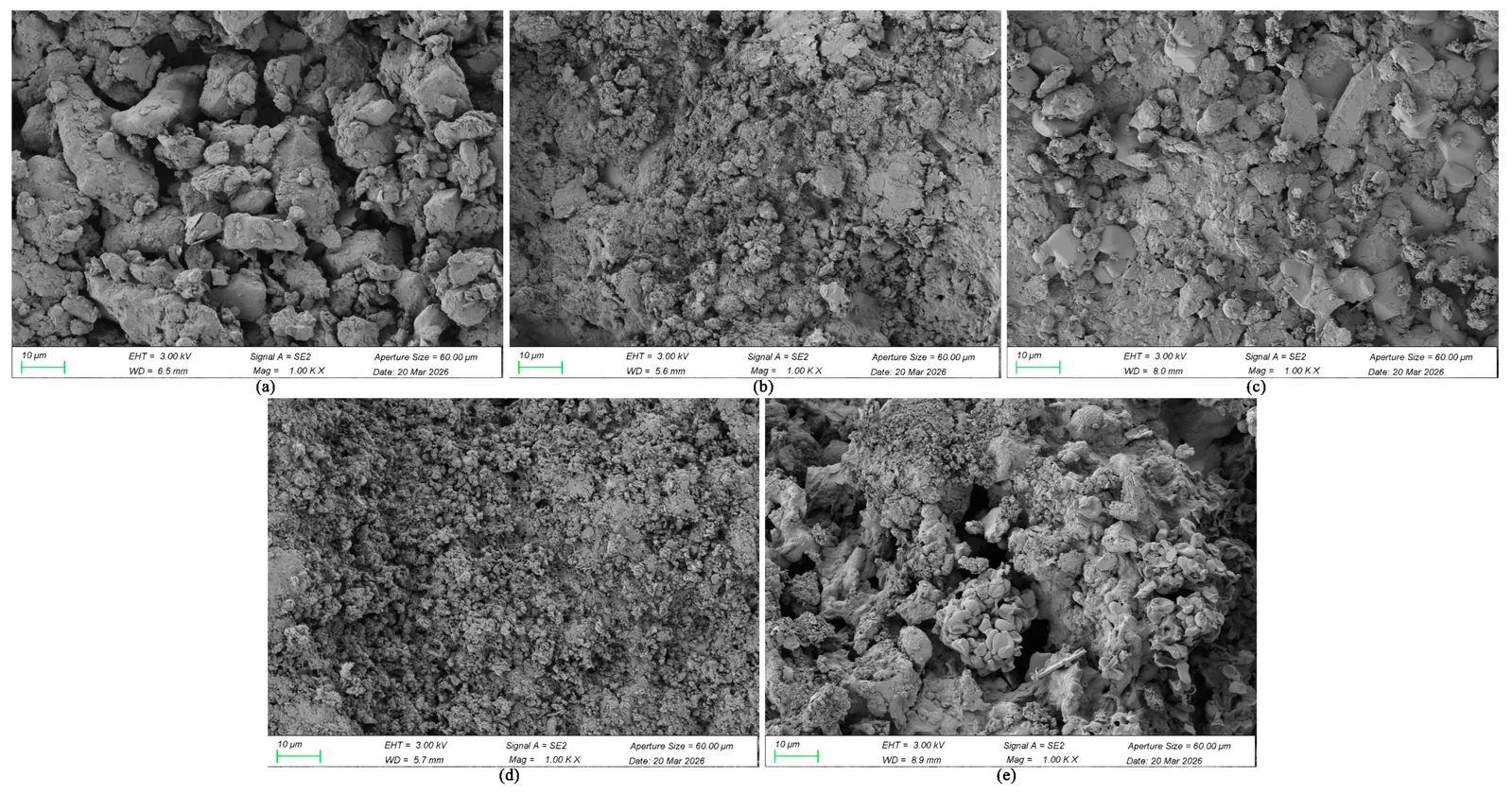
SEM secondary-electron images of Samples A-E at 1000×. (**a**) SEM morphology of particles in sample A; (**b**) SEM morphology of particles in sample B; (**c**) SEM morphology of particles in sample C; (**d**) SEM morphology of particles in sample D; (**e**) SEM morphology of particles in sample E. (Image source: compiled by the author based on SEM analysis results).

**Figure A10:**
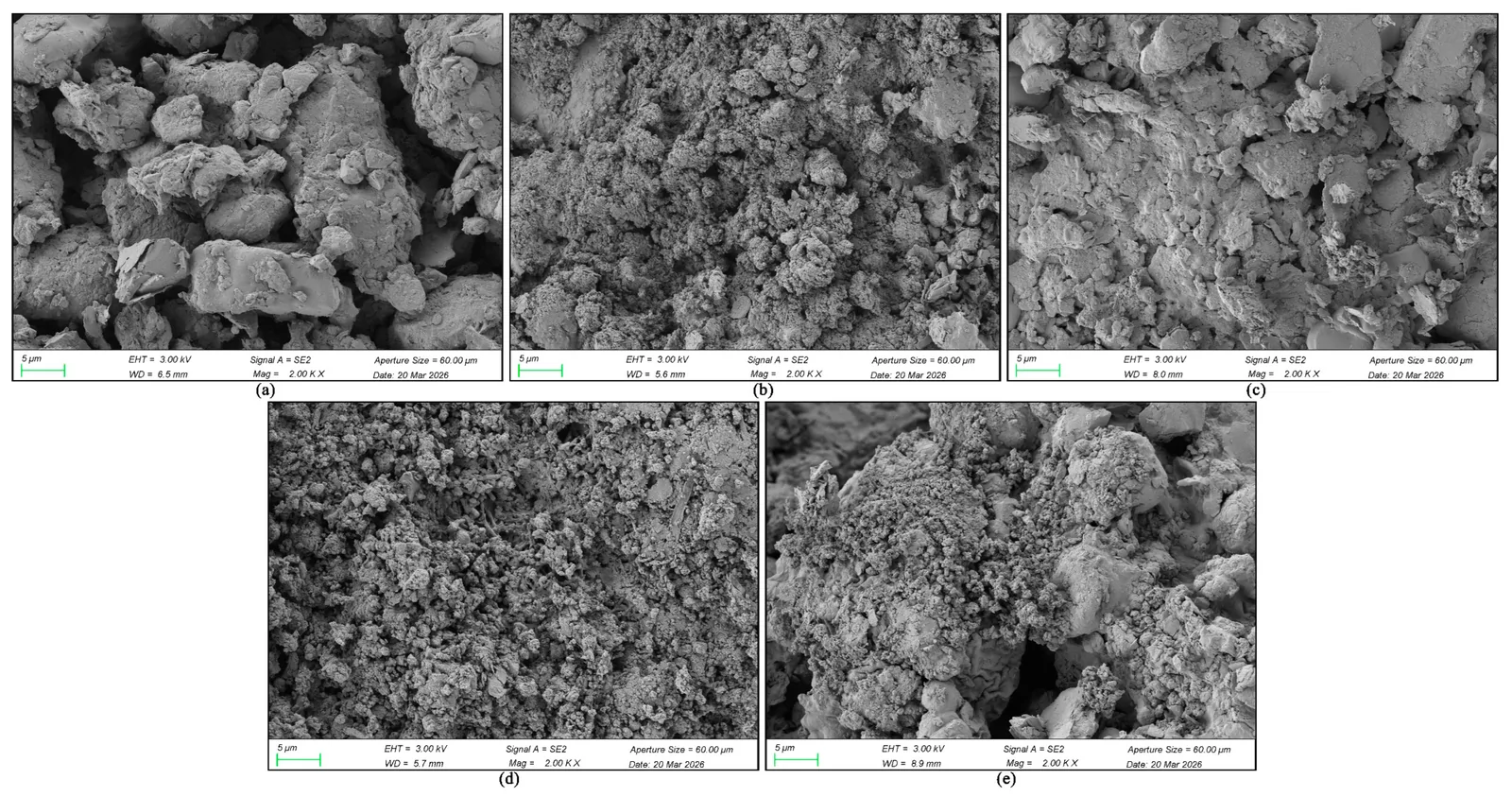
SEM secondary-electron images of Samples A-E at 2000×. (**a**) SEM morphology of particles in sample A; (**b**) SEM morphology of particles in sample B; (**c**) SEM morphology of particles in sample C; (**d**) SEM morphology of particles in sample D; (**e**) SEM morphology of particles in sample E. (Image source: compiled by the author based on SEM analysis results).

**Figure A11:**
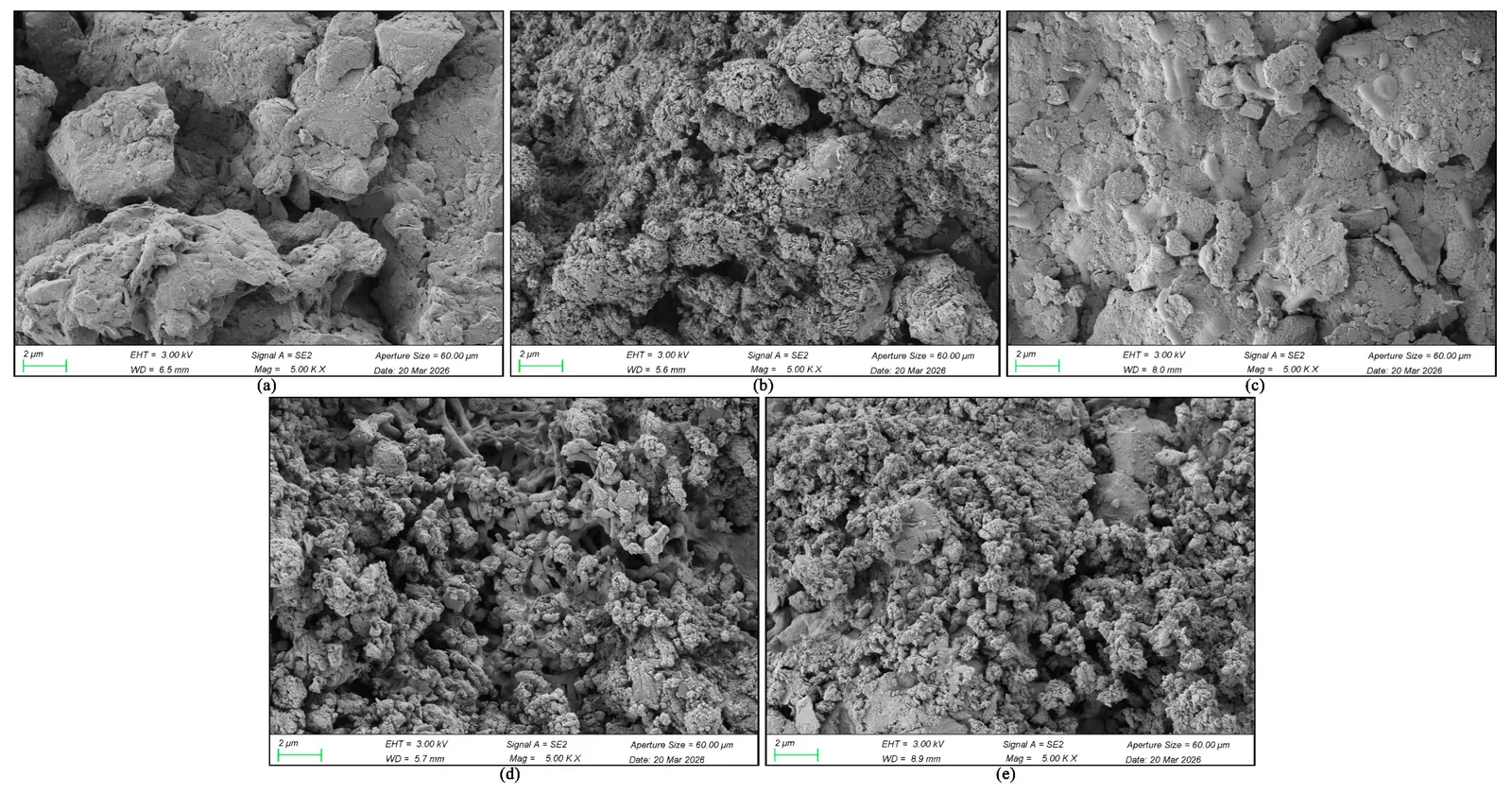
SEM secondary-electron images of Samples A-E at 5000×. (**a**) SEM morphology of particles in sample A; (**b**) SEM morphology of particles in sample B; (**c**) SEM morphology of particles in sample C; (**d**) SEM morphology of particles in sample D; (**e**) SEM morphology of particles in sample E. (Image source: compiled by the author based on SEM analysis results).

**Figure A13:**
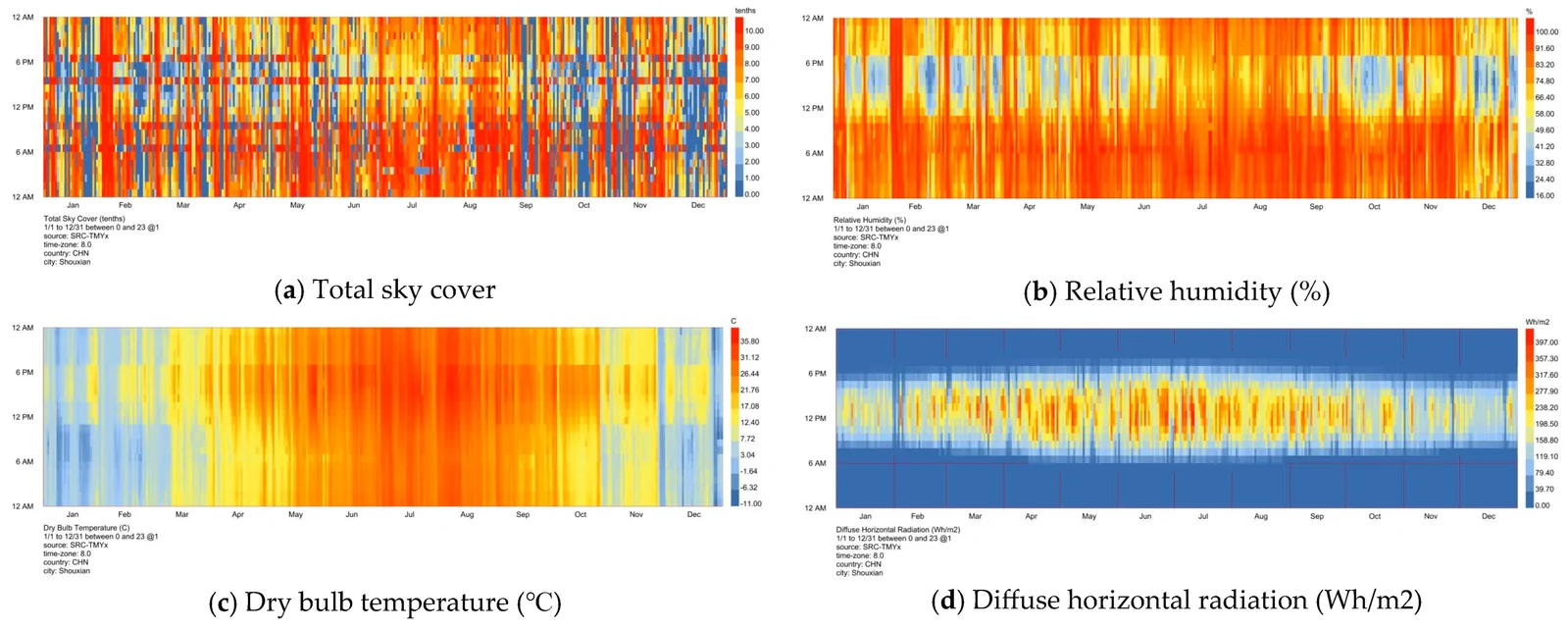

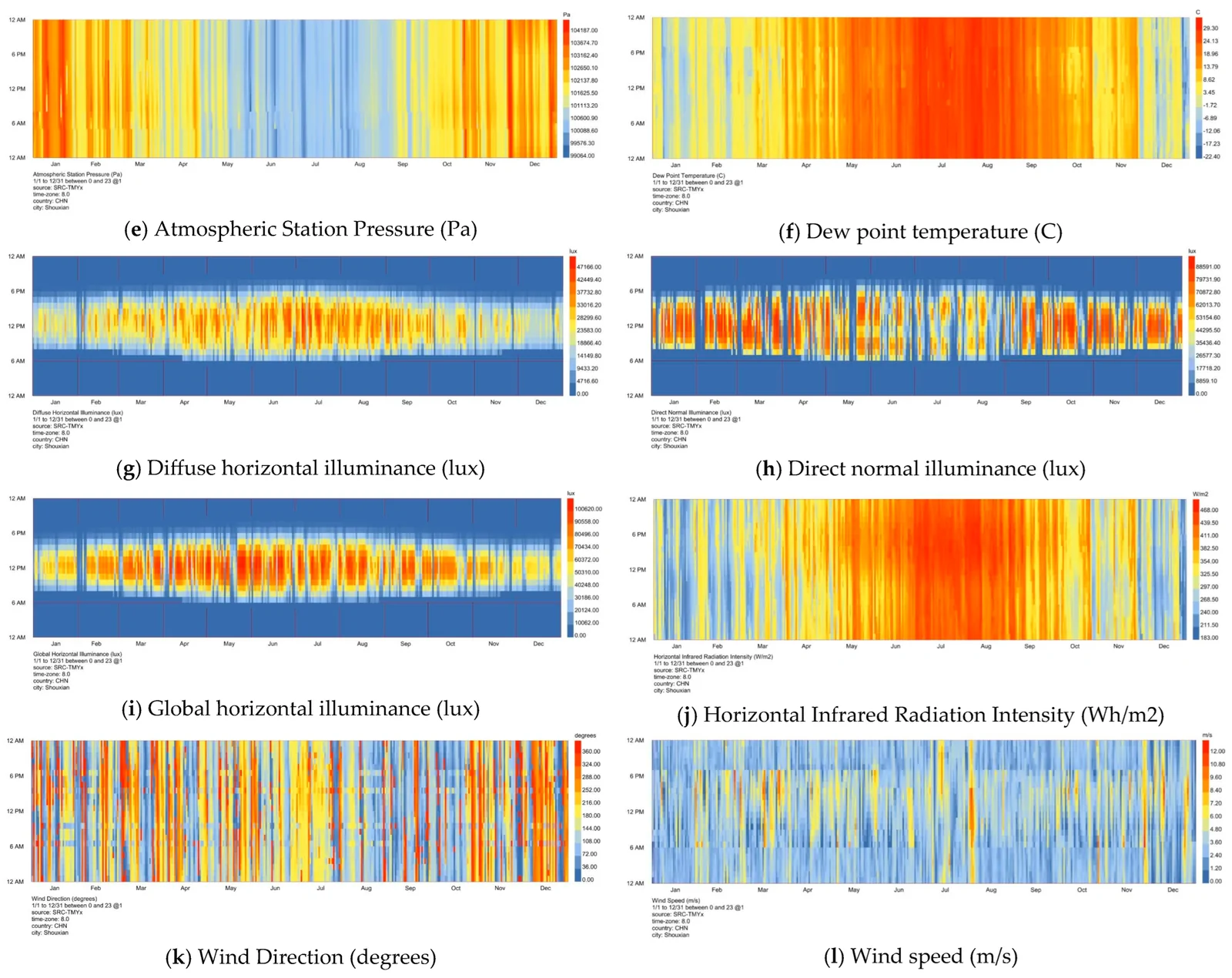
Climate analysis map in Shouxian County (Image source: by the author via Ladybug).

## Data Availability

The original contributions presented in this study are included in the article. Further inquiries can be directed at the corresponding authors.

## Appendix A. Four-Scale Petrographic Images of Six Thin Sections

Appendix A presents paired PPL and XPL images of the six thin sections at four image scales: 500, 200, 100, and 50 μm. The images show matrix–grain relationships, optical responses, and fractures. The polarization mode is indicated for each panel.

Sample A exhibits a matrix-supported overall structure at a 500 μm field of view, without a continuous coarse-grained framework (Figure A1). At 200 and 100 μm fields of view, a small number of bright-colored microcrystalline clusters are visible interspersed within a dark, fine-grained matrix, while the particle size distribution is relatively uniform at 50 μm. The continuity across all four scales supports classifying Sample A as a fine-grained matrix type.

Sample B exhibits a heterogeneous framework composed of large particles and dark intergranular regions at 500 μm, with some particles spanning different scale fields of view (Figure A2). Sub-angular boundaries and low-order interference colors are discernible at 200 and 100 μm, while at 50 μm, the fine matrix primarily fills the spaces between aggregates. These characteristics indicate that Sample B has a high aggregate content and poor sorting.

Sample C maintained a fine-grained, continuous matrix at 500 and 200 μm (Figure A3), with a few bright-colored clusters distributed within the matrix; the coarse-grained framework was not well-developed. Linear fractures penetrating the fine-grained matrix were more clearly visible at 100 and 50 μm. The petrographic texture of C is similar to that of A, with more clearly defined fractures and grain clusters.

Sample D-1 has a fine-grained matrix with few coarse grains (Figure A4). Weak to low-order interference colors occur across much of the section, together with small dark regions and narrow fractures.

Sample D-2 is dominated by a fine-grained matrix (Figure A5). Under plane-polarized light (PPL), the matrix is light-colored and translucent. Under cross-polarized light (XPL), most of the matrix shows weak birefringence, with sparse brighter grain clusters. This optical pattern is observed across the four image scales shown in Appendix A.

Sample E at 500 μm shows a mixture of various coarse-grained particles and a dark matrix (Figure A6), with significant differences in aggregate boundaries, interference colors, and internal particle structure. At 200 and 100 μm, low-grade gray-white interference color particles and higher interference color grains can be distinguished, and at 50 μm, the internal structure of the composite particles is clearer. Sample E exhibits the strongest mineral and particle size heterogeneity among the six thin sections.

## Appendix B. SEM Images at Other Magnifications

Appendix B presents SEM images of Samples A–E at 50×, 100×, 1000×, 2000×, and 5000×. The image series documents particle shape, aggregation, and local surface features across magnifications.

At a scale of 50× (Figure A7), samples A and C show a predominantly fine particle distribution with only a few larger particles, consistent with the fine-grained matrix structure in the thin section. Sample B exhibits a coexistence of coarse clumps and small particle areas, with noticeable undulations within the field. Sample D shows an interweaving of fine-grained coverage and blocky agglomerations. Sample E exhibits the greatest range in particle size and degree of agglomeration. Gaps are visible between the prepared particles.

At 100× magnification (Figure A8), the fine-particle spreading characteristics of sample A and sample C remain continuous, while large agglomerates and fine-particle regions coexist in sample B and sample E, and sample D exhibits blocky agglomerations covered by fine particles. This magnification connects the overall structure at low magnification with the particle boundaries at medium magnification.

At a 1000× scale (Figure A9), sample A shows irregular, blocky, and flaky particles covered with finer particles, with localized microcracks and lamellar shedding. Sample B exhibits a mix of plate-like, stepped fractures and irregular clusters, with significant differences between the two fields of view at the same magnification. Sample C contains both relatively smooth, rounded particles and coarse aggregates. Sample D shows the beginning of beaded and dendritic attachments on the surface of ordinary particles, while Sample E reveals lamellae edges and curled bud-like clusters, providing location information for high-magnification observation.

At 2000× magnification (Figure A10), the flaky and blocky particles of sample A, the stepped fracture of sample B, the smooth sliding particles of sample C, the dendritic attachments of sample D, and the lamellar edges of sample E were identified, showing that the five groups of samples had stable morphological differences from the middle magnification.

At 5000×, Sample D shows interwoven tubular features, whereas Sample E contains curled sheet-like objects (Figure A11).

## Appendix C. Detailed Data on EDS Mapping

Appendix C presents the software-reported wt%, wt% Sigma, and At% values for the five EDS-mapped regions shown in Figure 9, Figure 10, Figure 11, Figure 12 and Figure 13. Each region is reported separately in Figure A12, Table A1, Table A2, Table A3, Table A4 and Table A5.

The mapped region of A contains O, Si, Ca, and Na, together with Al, K, and other elements (Table A1).

**Table A1:** Detailed Data of Sample A on EDS Mapping.

Element	Line Type	Weight %	Weight % Sigma	Atomic %
C	K series	9.55	0.97	16.29
O	K series	38.51	0.56	49.30
Na	K series	7.12	0.17	6.35
Mg	K series	2.99	0.11	2.52
Al	K series	5.45	0.14	4.14
Si	K series	16.04	0.25	11.70
K	K series	2.77	0.12	1.45
Ca	K series	12.16	0.23	6.21
Ti	K series	0.92	0.14	0.39
Fe	K series	4.49	0.28	1.65
Total	/	100.00	/	100.00

Source: Statistical analysis by the author based on experimental results.

The mapped region of B has reported Fe and Si fractions of 62.96 and 4.51 wt%, respectively (Table A2).

**Table A2:** Detailed Data of Sample B on EDS Mapping.

Element	Line Type	Weight %	Weight % Sigma	Atomic %
C	K series	5.28	0.77	13.70
O	K series	20.45	0.32	39.80
Na	K series	0.17	0.13	0.23
Mg	K series	1.12	0.11	1.44
Al	K series	1.65	0.11	1.90
Si	K series	4.51	0.12	5.00
K	K series	0.28	0.08	0.22
Ca	K series	2.25	0.11	1.75
Ti	K series	1.33	0.13	0.86
Fe	K series	62.96	0.62	35.10
Total	/	100.00	/	100.00

Source: Statistical analysis by the author based on experimental results.

The mapped region of C contains O, Si, Ca, Al, and K (Table A3).

**Table A3:** Detailed Data of Sample C on EDS Mapping.

Element	Line Type	Weight %	Weight % Sigma	Atomic %
C	K series	11.22	0.57	19.58
O	K series	35.40	0.33	46.37
Na	K series	2.93	0.07	2.67
Mg	K series	1.27	0.05	1.09
Al	K series	7.58	0.09	5.89
Si	K series	17.10	0.16	12.75
K	K series	4.72	0.08	2.53
Ca	K series	11.33	0.13	5.92
Ti	K series	0.46	0.08	0.20
Fe	K series	7.99	0.19	3.00
Total	/	100.00	/	100.00

Source: Statistical analysis by the author based on experimental results.

O, Ca, and C are the principal elements reported for the mapped region of D (Table A4).

**Table A4:** Detailed Data of Sample D on EDS Mapping.

Element	Line Type	Weight %	Weight % Sigma	Atomic %
C	K series	21.83	0.84	33.69
O	K series	40.27	0.67	46.62
Na	K series	0.76	0.14	0.62
Mg	K series	3.49	0.14	2.66
Al	K series	0.74	0.12	0.51
Si	K series	4.54	0.14	3.00
K	K series	0.53	0.11	0.25
Ca	K series	26.05	0.42	12.05
Ti	K series	0.00	0.18	0.00
Fe	K series	1.79	0.34	0.60
Total	/	100.00	/	100.00

Source: Statistical analysis by the author based on experimental results.

C and O are the principal elements reported for the mapped region of E (Table A5).

**Table A5:** Detailed Data of Sample E on EDS Mapping.

Element	Line Type	Weight %	Weight % Sigma	Atomic %
C	K series	49.22	0.40	59.28
O	K series	40.15	0.37	36.32
Na	K series	0.32	0.06	0.20
Mg	K series	0.69	0.05	0.41
Al	K series	0.22	0.05	0.12
Si	K series	2.58	0.06	1.33
K	K series	0.79	0.06	0.29
Ca	K series	4.82	0.10	1.74
Ti	K series	0.01	0.08	0.00
Fe	K series	1.20	0.16	0.31
Total	/	100.00	/	100.00

Source: Statistical analysis by the author based on experimental results.
